# Synergistic adsorption and oxidation of arsenite by Fe–Mn binary oxide-modified bamboo biochar in the presence of air

**DOI:** 10.1038/s41598-025-32178-5

**Published:** 2025-12-27

**Authors:** Omar Rady, Ahmed Bakr, Mohamed G. Moussa, Belal Nodhy

**Affiliations:** 1 Soils and Water Department, Faculty of Agriculture, Al-Azhar University, Cairo, 11651 Egypt; 2 Environment and Bio-Agriculture Department, Faculty of Agriculture, Al- Azhar University, Cairo, 11651 Egypt; 3 Soil and Water Research Department, Nuclear Research Center, Egyptian Atomic Energy Authority, Cairo, 13759 Egypt; 4 Central Laboratory for Environmental Quality Monitoring (CLEQM), National Water Research Center (NWRC), El Qanater, Egypt

**Keywords:** Arsenic, Biochar, Fe-Mn binary oxide, Adsorption, Oxidation, Chemistry, Environmental sciences, Materials science

## Abstract

The adsorption and oxidation processes of arsenite (As(III)) are investigated in this work utilizing bamboo biochar (BC) modified with Fe, Mn, and Fe-Mn binary oxides (FBC, MBC, FMBC). The modification resulted in substantial enhancements to the bamboo biochar’s surface area, pore structure, and functional groups. Batch adsorption experiments showed that FMBC achieved the highest As(III) removal capacity, reaching 5.86 mg g^− 1^, outperforming both FBC and MBC. The kinetics data were fitted with a pseudo second-order model and analyzed using the Langmuir-Freundlich isotherm model, resulting in a good correlation coefficient. Characterization techniques, including XRD, FTIR, XPS, and BET analysis, confirmed successful loading of metal oxides and demonstrated As(III) oxidation to arsenate (As(V)) during adsorption, particularly under oxic conditions. Manganese oxides played a pivotal role in catalyzing the oxidation of As(III), whereas iron oxides predominantly facilitated the adsorption of arsenic (As) species. pH and coexisting anions were found to influence As removal, with FMBC maintaining high performance across a broad pH range. Additionally, the presence of dissolved oxygen (DO) enhanced As(III) oxidation, improving overall removal efficiency. The synergistic action of the Fe-Mn oxides significantly enhanced both the rate and extent of As(III) oxidation, a critical step for subsequent adsorption. These combined features position FMBC as an efficient, cost-effective, and highly promising functionalized biochar for As removal.

**Supplementary Information:**

The online version contains supplementary material available at 10.1038/s41598-025-32178-5.

## Introduction

As is a common element in sediments, soils, and waters. Its compounds have toxic and carcinogenic effects on human health^1,2^. In soil-water systems, pentavalent arsenate (As(V)) and trivalent arsenite (As(III)) are two common inorganic oxyanions. As(III) is more mobile, soluble, and toxic than As(V), making it more challenging to remove from water^3,4^. Furthermore, The biogeochemical cycling of arsenic is highly sensitive to microbial activity, pH, and the oxidation-reduction potential (Eh) within soils and water^1^. As(III) oxidation and adsorption are two crucial steps in developing technology to remove As from the environment^5^.

Materials that include carbon, such as graphene, biochar, and carbon nanotubes, are especially promising for effectively adsorbing heavy metals because of their large specific surface area and porous nature^6^. Biochar is a carbon-rich solid material derived from the thermochemical decomposition of biomass under limited or no oxygen conditions, typically through pyrolysis of agricultural residues, forestry waste, or other organic by-products. Recently, biochar has drawn attention from researchers because of their plentiful sources, significant porosity, large surface area, strong chemical stability, and variety of functional groups (such as carboxyl, hydroxyl, and phenolic groups)^7^. However, because of its small number of surface positive charge sites, pristine biochar typically has a very low adsorption capacity for hazardous oxyanions^7,8^. In this case, Several modifications have been suggested to raise the biochar’s positive surface charges, which will improve its ability to adsorb As, such as Zn-Fe oxides^9^, Fe_3_O_4_^10^, and Fe-Mn binary oxides^11^. It is one of the most promising methods for As mitigation using metal binary oxides such as modified biochar, due to its relative simplicity, low cost, low energy consumption and low waste^12^. Fe-Mn binary oxides exhibit enhanced As(III) removal efficiency due to the synergistic interaction between Mn oxides, which act as oxidizing agents, and Fe oxides, which serve as effective adsorbents^13^. Although Fe–Mn binary oxide-modified biochar has been explored as an effective adsorbent for arsenic removal^3,14^, the underlying mechanisms governing As(III) adsorption and oxidation under varying environmental conditions remain insufficiently understood. In particular, the role of Fe and Mn oxides in bamboo-derived biochar under different atmospheric conditions has not been systematically investigated.

Accordingly, the purposes of this work were as follows: (i) to prepare, describe, and assess the As(III) removal performance of MBCs in three species (Fe, Mn and Fe-Mn binary oxide); (ii) to examine the oxidation and adsorption kinetics of As(III) on MBCs; and (iii) to describe how As(III) is adsorbed and oxidized by MBCs. The impact of DO and pH on the chemical pathways was also investigated.

## Materials and methods

### Preparation of Fe-Mn oxide modified bamboo biochars

This study prepared BC from bamboo (*G. chacoensis clumps*). Fragments (~ 45 cm from the base) were cut from the discarded young growth. Producers remove these culms due to their low economic value and to promote the expansion of the clumps. The collected culms were cleaned to eliminate contaminants and then dried at 70 °C for 5 h. A slow pyrolysis process was performed on dried and cleaned culms, followed by pyrolysis at 700 °C for 8 h, followed by a cold decline to 25 °C. Finally, the generated BC was crushed and stored in a plastic container. A FeSO_4_·7H_2_O solution (80 mL, 0.24 mol L^− 1^) was mixed with 5 g BC to create FBC. H_2_O_2_ was then gradually added (30%, 0.4 mL min^− 1^) to the FeSO_4_·7H_2_O solution at a ratio of 1:0.5. After six hours of stirring with a magnetic stirrer, the suspension was filtered and allowed to dry overnight at 70 °C^15^. FMBC was prepared by adding 5 g BC into FeSO_4_·7H_2_O (40 mL, 0.20 mol L^− 1^) and KMnO_4_ (40 mL, 0.10 mol L^− 1^) solutions. Additionally, the suspension was continuously stirred for 6 h and dried at 95 °C. The mixture was pyrolyzed once more at 600 °C for 0.5 h in a nitrogen environment after being ultrasonically dispersed for two hours and dried in a water bath at 95 °C^16^. The last modification method was MBC. After soaking in 80 mL of KMnO_4_ solution (0.15 mol L^− 1^), BC (5 g) was dried in an oven at 70 °C for the whole night and then pyrolyzed once more at 600 °C for 0.5 h in a nitrogen atmosphere^17^.

### Batch As(III) adsorption experiments

Adsorption batch studies were conducted to investigate the impact of different MBCs on As(III) adsorption capabilities. At different initial concentrations (0–60 mg L^–1^), 30 mL of As(III) solutions were mixed with 1 g L^–1^ of BC, MBC, FBC, and FMBC. The pH value of the reaction system was kept steady at 7.0 by using both diluted NaOH and HNO_3_ at intervals, and the ionic strength was maintained using a 0.01 M NaNO_3_ solution. After 24 h of agitation (200 rpm, room temperature), the samples were centrifuged for 5 min at 9000 rpm, and 0.1 M NaOH solution desorbed the adsorbed As from the solid products over 2 h. The quantities of dissolved total As, As(III), and As(V) were measured by filtering the supernatants using 0.22 μm membranes following varying reaction times. The As(III) adsorption performance in the solution was calculated by decreasing the As(III) concentration after the reaction. A cyclic experiment was conducted to evaluate the practical viability of modified bamboo biochar adsorbents. Specifically, 1 g L^–1^ of MBC, FBC, and FMBC were added to a 15 mg L^–1^ As(III) solution, followed by magnetic stirring at 500 rpm for 1440 min. The samples were then collected for analysis. The solution was filtered to separate the solid particles, which were subsequently dried in a drying oven at 60 °C. Regeneration experiments were performed using a 0.1 M NaOH solution as the eluent, stirred for 120 min. The desorption experiment was repeated four times. A parallel experiment was conducted to calculate the experimental error.

### As(III) adsorption and oxidation

The link between the two-phase interface’s adsorption capacity and equilibrium concentration was demonstrated using adsorption isotherms. For isotherm experiments, batch adsorption experiments were conducted under different temperatures. As(III) solutions with initial concentrations ranging from 0 to 60 mg L^–1^ were prepared, and 20 mL of the solution was placed in polyethylene vials with 0.02 g of BCs. The experiments were carried out using the protocol described in previous batch experiments. We collected and kept the modified and separated BCs in a vacuum bag. The adsorption capacity of As on BC were calculated as following equations:

1 $${q_e}=\frac{{As{{(III)}_{ads}}+As{{(V)}_{ads}}}}{m}$$

Here, q_e_, As(III)_ads_, and As(V)_ads_ are the adsorption capacity of As, adsorbed As(III) and adsorbed As(V) on BC, respectively.

Adsorption kinetics is a crucial metric for characterizing both chemical and heterogeneous catalytic adsorption. Approximately 15 mg L^− 1^ As(III) solution was introduced to 1 g L^− 1^ BC, FBC, MBC, and FMBC in glass reaction containers under nitrogen and air atmospheres. HNO_3_ (0.01 mol L^− 1^) and NaOH (0.01 mol L^− 1^) were added to bring the pH down to 7.0, and the mixture was stirred for 24 h at 25 °C as extensively described in our earlier study^1^. Samples were collected and filtered to determine the concentrations of Mn and Fe at different time intervals (0, 120, 240, 360, 480, 720 and 1440 min).

Two comparable experiments were conducted to study the effect of pH on As(III) oxidation and adsorption at pH 5.0 and 9.0. To explore the influence of coexisting ions, 5 m mol L^− 1^ of HCO_3_^−^, SiO_3_^2−^, SO_4_^2−^ and PO_4_^3−^ were adjusted to study their effects on the As(III) adsorption performance. 1 g L^–1^ of BCs was added to 20 mL of 15 mg L^–1^ As(III). A parallel experiment was repeated to calculate the experimental error.

### Analysis

The adsorbents’ structure, chemical composition and surface charges were investigated by XRD, FTIR, XPS, BET and PZC. Information on characterization and chemical analysis is provided in the supplementary material. Parallel experiments were performed to calculate the standard deviation.

## Results

### Characterization of modified biochars

Table 1 presents the elemental composition, surface area, and textural properties of the synthesized biochar samples. The total carbon (C) contents were 73.29, 55.41, 62.53 and 53.82% and that of hydrogen (H) were 6.41, 3.42, 4.86 and 3.22% for BC, MBC, FBC and FMBC, respectively. The molar ratios of hydrogen to carbon (H/C) were 0.08, 0.06, 0.07 and 0.05 for BC, MBC, FBC and FMBC, respectively. The degree of carbonization, indicated by the H/C molar ratio, reflects the aromaticity and structural maturity of biochar. In this study, the modified biochars showed lower H/C ratios than pristine BC, confirming a higher degree of carbonization and greater aromatic stability^18^. The pristine biochar (BC) exhibited a low specific surface area (60.2 m^2^ g^− 1^), which increased markedly after Fe–Mn modification to 119.32, 121.72, and 163.26 m² g^− 1^ for MBC, FBC, and FMBC, respectively (Table 1).

**Table 1:** Physicochemical characteristics of BC and modified BCs.

Samples	Bulk elemental composition (%)				Ash content (%)	S_BET_ (m^2^ g^− 1^)	V_tot_ (cm^3^ g^− 1^)
	C	H	S	N			
BC	73.29	6.41	0.00	1.55	32	60.2	0.025
MBC	55.41	3.42	0.00	1.47	27.52	119.32	0.062
FBC	62.53	4.86	0.36	1.6	61.31	121.46	0.064
FMBC	53.82	3.22	0.31	1.54	36.6	163.26	0.102

The incorporation of Fe and Mn oxides effectively activated and expanded the pore structure of biochar, enhancing surface accessibility and adsorption capacity. This improvement could be attributed to KMnO₄-induced transformation of nanopores into mesopores or macropores, resulting in increased total pore volume and specific surface area^16^.

**Fig. 1:**
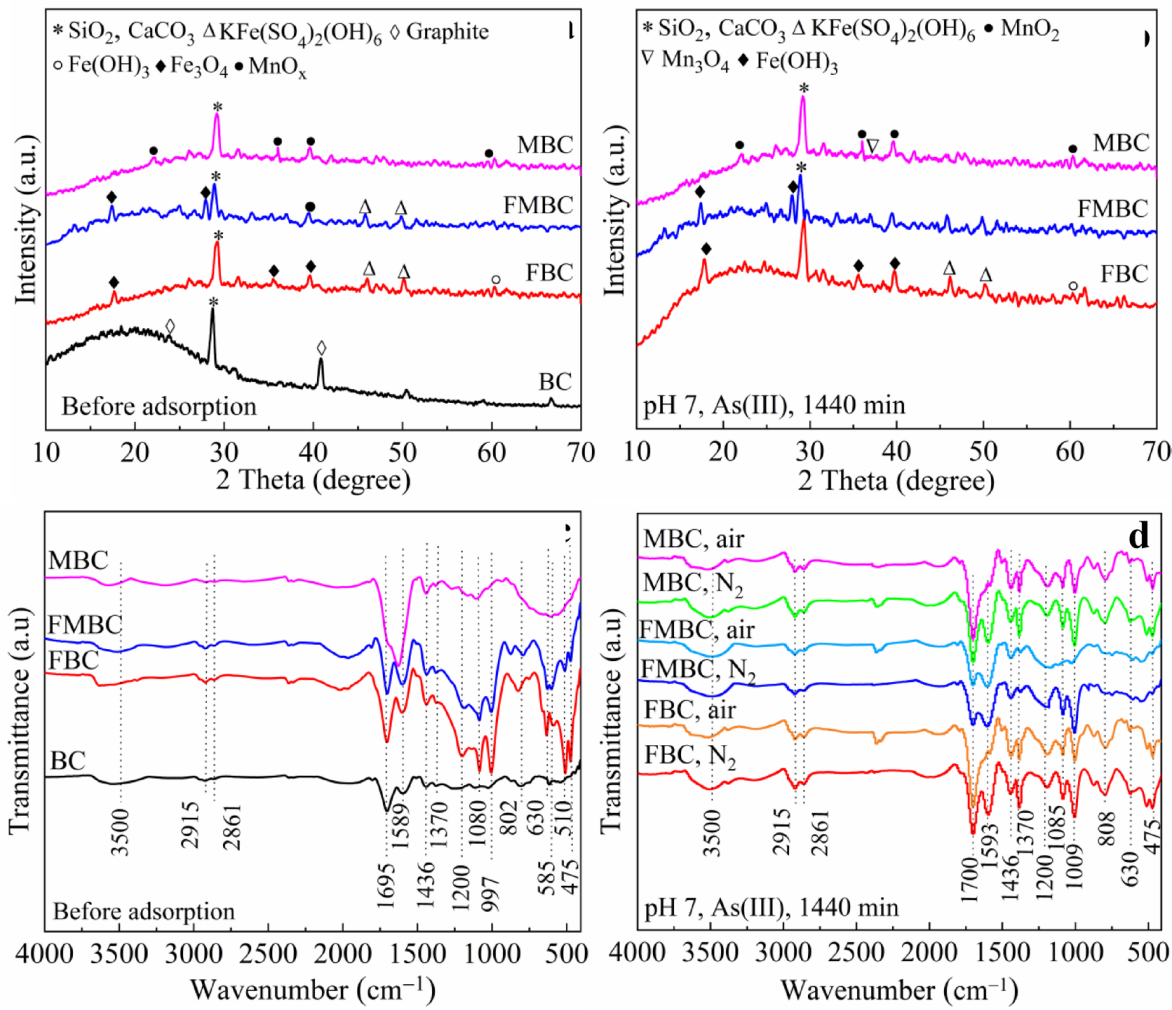
XRD pattern (**a** and **b**) and FTIR spectra (**c** and **d**) of modified BCs before and after reaction with 15 mg L^–1^ As(III) at pH 7.0 under different atmospheres.

The crystalline structures of the modified biochars before and after As(III) adsorption were characterized using X-ray diffraction (XRD) analysis (Fig. 1(a) and (b)). The XRD pattern of pristine BC displayed distinct graphite peaks at 2θ = 23.9° and 41.0°, and a diffraction peak at 28° attributed to SiO₂ and CaCO₃ phases. Following the modification, the intensities of these peaks markedly decreased. In the spectra of FBC and FMBC, the peaks at 2θ = 17.8°, 35.1°, and 39.2° were associated with the presence of Fe₂O₃, whereas the peak at 60.7° corresponded to the Fe(OH)₃ phase^19,20^. The spectrum of MBC showed fewer diffraction peaks than BC, demonstrating that Mn oxide was successfully loaded into BC. The weak diffraction peaks were observed at 22.0°, 36.8° and 42.0°, which were attributed to the presence of Mn oxide in two modified BCs^21^. The earlier findings indicated that the impregnation process may have an impact on the materials’ crystallinity. FTIR analysis was used to identify the surface functional groups of modified BCs. As shown in Fig. 1(c), The FTIR spectra of all biochars exhibited distinct absorption bands at approximately 3484, 2900, and 1700–1595 cm^− 1^, corresponding to − OH stretching vibrations of phenolic and alcoholic groups, aliphatic − C−H stretching, and carbonyl (C = O) vibrations, respectively. Additional bands at 2850 and 1433 cm^− 1^ were assigned to aliphatic methylene/methyl (− CH₂/−CH₃) groups and carboxylic (− COOH) vibrations, respectively, indicating the presence of diverse oxygen-containing functional groups on the biochar surfaces^8^. C = O stretching vibrations in ketones and aldehydes, as well as the coupling between benzene-ring skeletons (C = C) and asymmetric carboxyl vibrations, were identified as the causes of the bands at 1698 and 1589 cm^− 1^, respectively^22^. A strong band located at 1000–1150 cm^− 1^ indicated the presence of alcohols and carboxylic acids, esters and ethers, and other bands at 1100–1320 cm^− 1^ were also evident. FBC and FMBC had a band at 1080 cm^− 1^, indicating that Fe − OH was immobilized on the biochar^23^. After modification with Fe-Mn binary oxide, there were two apparent peaks at 475 and 585 cm^− 1^, attributed to the presence of Mn − O and Fe − O groups, respectively^24^. These findings further demonstrated the presence of Fe and Mn oxides. The crystallinity and structural properties of the modified bamboo biochars (mBCs) were also unaffected by As(III) adsorption following a reaction of 1440 min.

**Fig. 2:**
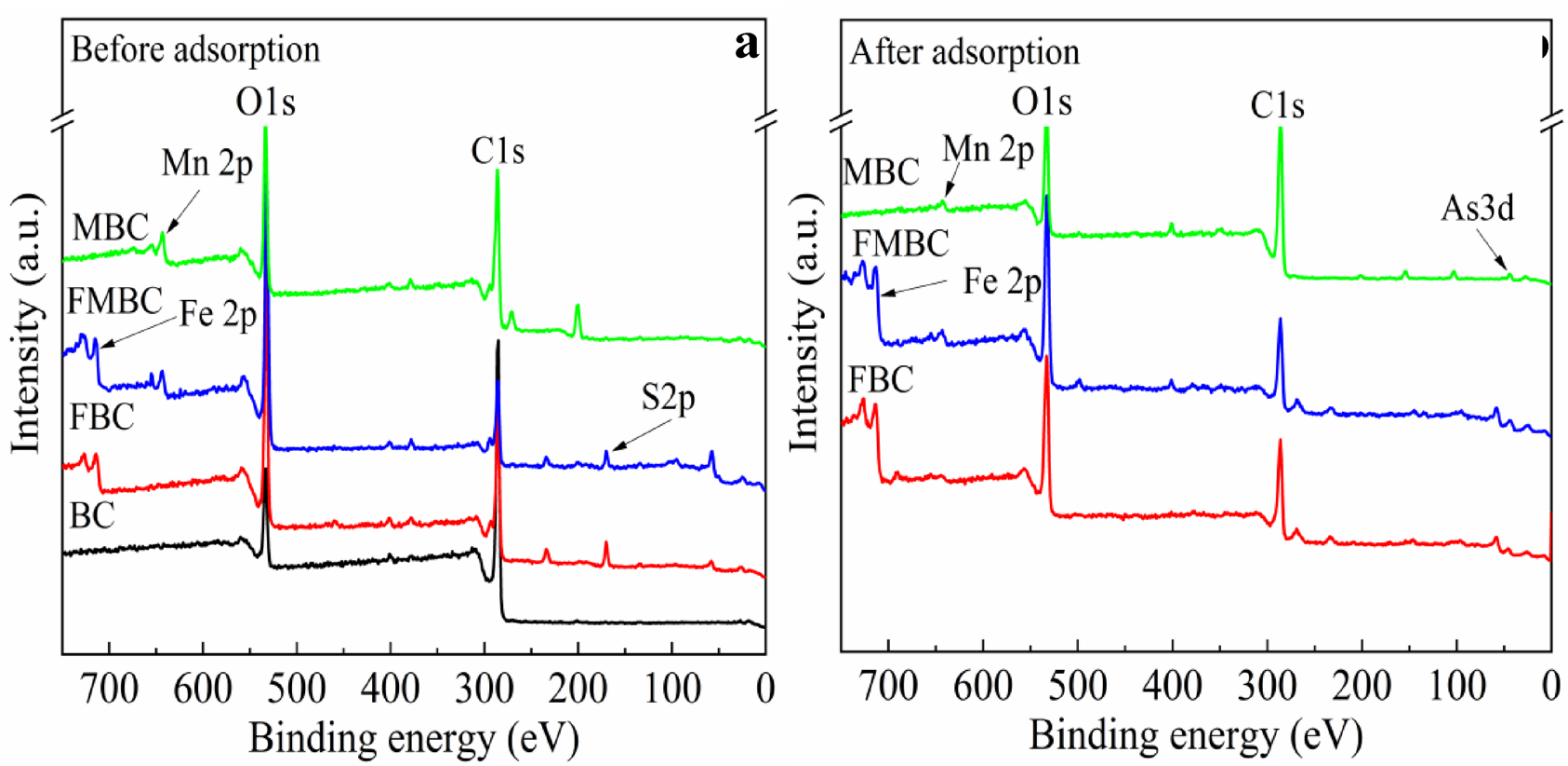
Full-scan XPS spectra of modified BCs before (**a**) and after As(III) adsorption (**b**).

XPS investigated the elemental composition and oxidation states of the modified BCs regions before and after As(III) adsorption. The presence of inorganic oxygen linked to the metal oxides of the modified BCs was demonstrated by the large variation of the O 1 s peaks (Fig S1). The binding energy peaks at 529.50 and 530.72 eV correspond to metal-oxygen bonds (M-O) and metal-hydroxyl bonds (M-OH), respectively (Fig. S1(a-c)). Three compounds are present in modified BCs, as shown by the deconvolution of the C 1 s spectra (see supplementary material), which may be split into three separate peaks. The binding energies for -COOH, C-O-C/C-H, and C-O-C/C-H are 288.96, 285.65, and 284.63 eV, respectively (Fig. S1(d-f))^16^. XPS analysis was used to identify the binding energy transfers of the elements As, Mn, and Fe; the findings are illustrated in Fig. 2. Moreover, Fig. 2a shows the Full The XPS survey spectra confirmed that pristine and modified biochars were mainly composed of Si, O, Fe, and Mn, verifying the successful incorporation of Fe–Mn oxides. Pre-adsorption analysis showed no detectable As signals on the adsorbent surface. However, the subsequent appearance of defined As 3 d photoelectron peaks provided direct spectroscopic evidence that Fe-Mn oxide active sites facilitated arsenic sequestration through coupled surface complexation and redox interactions (Fig. 2b). In agreement with the FTIR study, the XPS examination showed that Fe and Mn oxides precipitated on BC. This result also confirmed that As was successfully adsorbed on the surface of modified BCs.

One of the most important adsorbents properties to ascertain is the point of zero charge (PZC). The zeta potential of FBC and FMBC decreased with increasing pH. The PZC value (pH_pzc_) is the corresponding pH value when each surface charge is equal (Fig. S2). The PZC values were 7.20 and 6.75 for FBC and FMBC, respectively. The zeta potential was positive when the pH was lower than pH_pzc_, which was desirable for anion adsorption via electrostatic attraction. The zeta potential decreased with increasing pH; the anions’ adsorption was difficult. Conversely, both MBC and BC showed negative zeta potential across the wide pH range, indicating that their surfaces remained predominantly negatively charged. Moreover, the zeta potential values decreased sharply between pH 2.0 and 4.0, after which no significant variation was observed.

### Study of As(III) adsorption

**Fig. 3:**
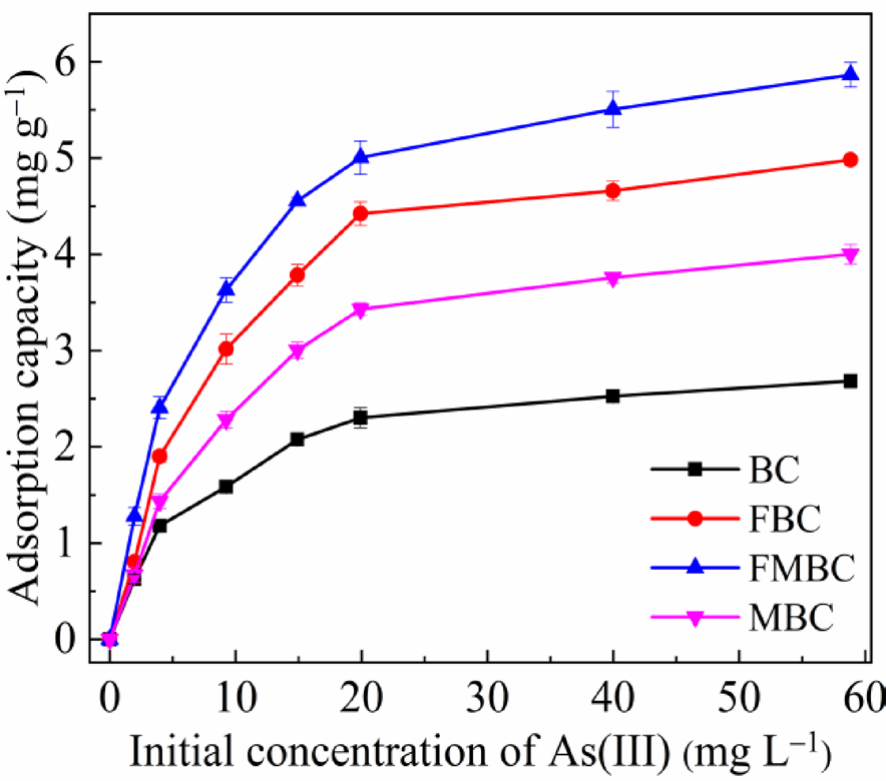
Adsorption capacities of As(III) in the reaction systems containing initial concentrations of As(III) and 1.0 g L^− 1^ BC and modified BCs at pH 7.0.

The effect of the initial concentration on the As(III) adsorption was evaluated at pH 7.0 for BC within 1440 min at 25 °C (Fig. 3). Adsorption capacities of As(III) were biphasic, with a rapid initial adsorption phase, followed by a slower phase in the subsequent time. The adsorbed As increased sharply and gradually became stable to reach equilibrium when the initial As(III) concentration was 15 mg L^− 1^. Notably, the As adsorption capacities by MBC, FBC and FMBC separately were 4.00, 4.98 and 5.86 mg g^− 1^, which were remarkably higher than those of BC at 2.68 mg g^− 1^. As for FMBC had an adsorption capacity almost 2.18 times higher than BC.

These results suggested that the improved As(III) removal by modified BCs was attributed to the higher porosity and the existence of Fe and Mn oxides. Additionally, As adsorption on negatively charged BC surfaces could be inhibited by electrostatic repulsion. Furthermore, the negative charges left on BCs by delayed pyrolysis prevented them from adsorbing anions^25^.

**Fig. 4:**
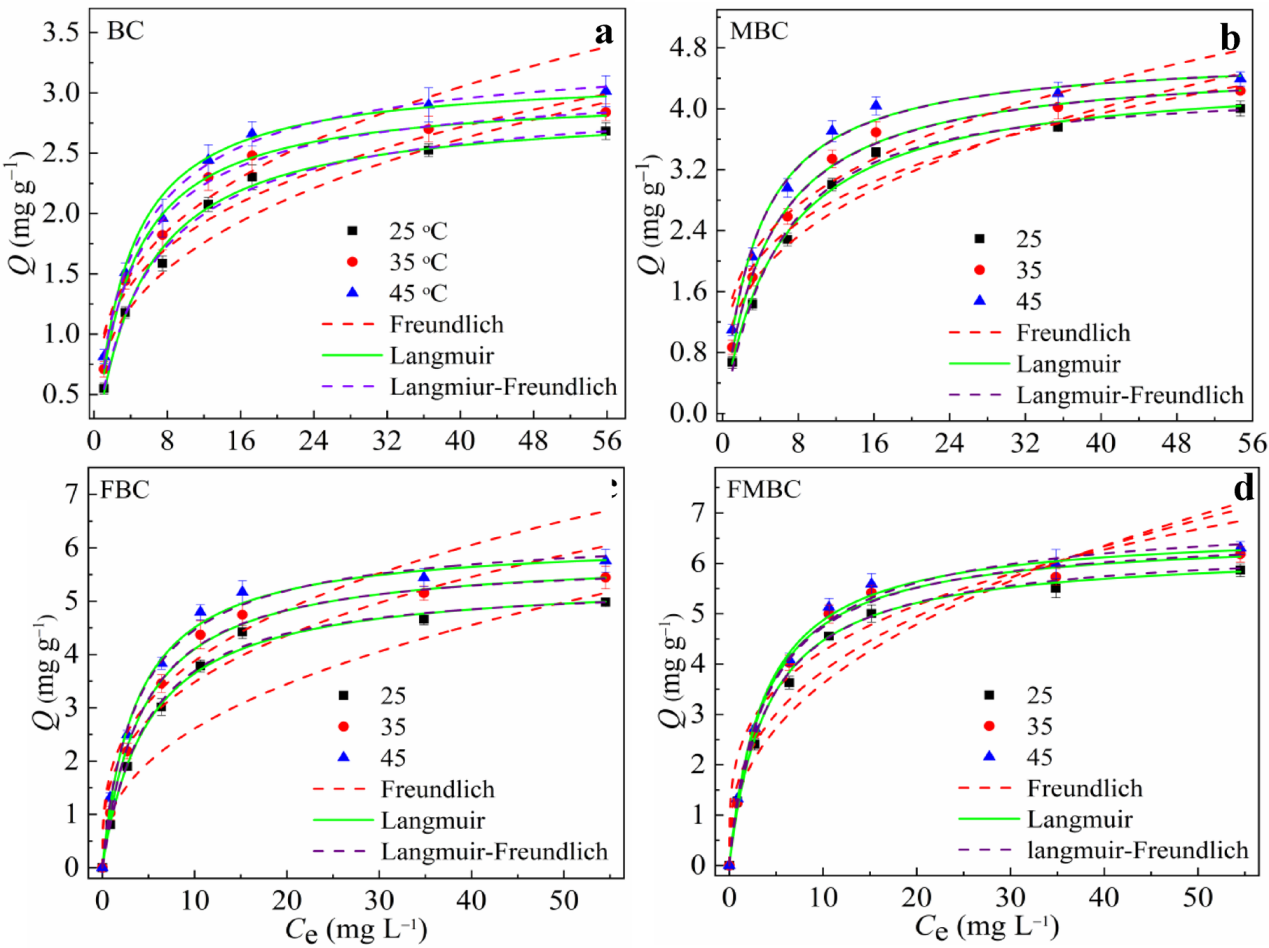
Adsorption isotherm data and modeling for As(III) on BC and modified BCs at different temperatures (initial pH: 7.0; adsorbent dosage: 1 g L^− 1^; contact time: 1440 min).

Figure 4; Table 2 present the isotherm analysis of As(III) adsorption on BC and its modified forms (FBC, FMBC, and MBC), showing that adsorption increased with temperature, confirming an endothermic process consistent with the Langmuir, Freundlich, and Langmuir–Freundlich models.

**Table 2:** Adsorption isotherm models parameters for As(III) adsorption onto BC and modified BC.

Samples	T(°C)	Freundlich model			Langmuir model				Langmuir-Freundlich model			
		*k*_F_	*1/n*	*R* ^2^	*q*_m_		*k*_L_	*R* ^2^	*q*_m_	*k*_L-F_	*1/n*	*R*_2_
BC	25	0.77	0.33	0.938	2.89		0.19	0.994	3.02	0.20	0.91	0.994
	35	0.95	0.29	0.936	3.00		0.27	0.995	3.11	0.28	0.89	0.996
	45	0.98	0.31	0.932	3.16		0.26	0.993	3.40	0.29	0.85	0.996
	25	1.15	0.33	0.909	4.48		0.16	0.992	4.28	0.14	1.12	0.994
MBC	35	1.39	0.28	0.940	4.61		0.20	0.997	4.63	0.20	0.98	0.997
	45	1.50	0.28	0.899	4.72		0.27	0.994	4.74	0.27	0.98	0.994
	25	1.00	0.40	0.983	5.44		0.20	0.999	5.38	0.20	1.03	0.999
FBC	35	1.65	0.32	0.984	5.86		0.23	0.999	6.66	0.26	0.97	0.999
	45	1.69	0.35	0.974	6.69		0.26	0.998	7.01	0.26	0.91	0.999
	25	2.23	0.27	0.990	6.26		0.24	0.992	6.44	0.25	0.94	0.999
FMBC	35	1.41	0.40	0.973	6.55		0.26	0.999	6.65	0.26	0.97	0.994
	45	1.69	0.35	0.974	6.69		0.26	0.998	7.01	0.26	0.91	0.999

In the Freundlich model, chemisorption was the primary mode of As adsorption onto all adsorbents because the *1/n* ratio was < 1. In the Langmuir model at 25 °C, the *q*_*m*_ of As for FMBC (6.26 mg. g^− 1^) was about 1.15, 1.39, and 2.16 times greater than that of FBC (5.44 mg. g^− 1^), MBC (4.48 mg. g^− 1^) and BC (2.89 mg. g^− 1^), respectively.

The adsorption energies were described by the Langmuir constant (*k*_*L*_), which were 0.19, 0.16, 0.20 and 0.24 L mg^− 1^ for BC, MBC, FBC and FMBC, respectively. The correlation coefficients (*R*^*2*^) values of the Freundlich model were in the range of 0.899–0.999, whereas those of the Langmuir and Langmuir-Freundlich models were in the range of 0.992–0.999 for all adsorbents at different temperatures. Based on the previous results and analysis, the Langmuir and Langmuir-Freundlich models fit the data better than the Freundlich model. The Langmuir-Freundlich model was suitable to explain the adsorption mechanism of As(III) on all BCs. Adsorption increased at lower As(III) concentrations, whereas monomolecular adsorption occurred at higher concentrations. A similar finding for Cr adsorption on biochar was observed in Mohan, et al.^20^.

### As adsorption and oxidation kinetics

**Fig. 5:**
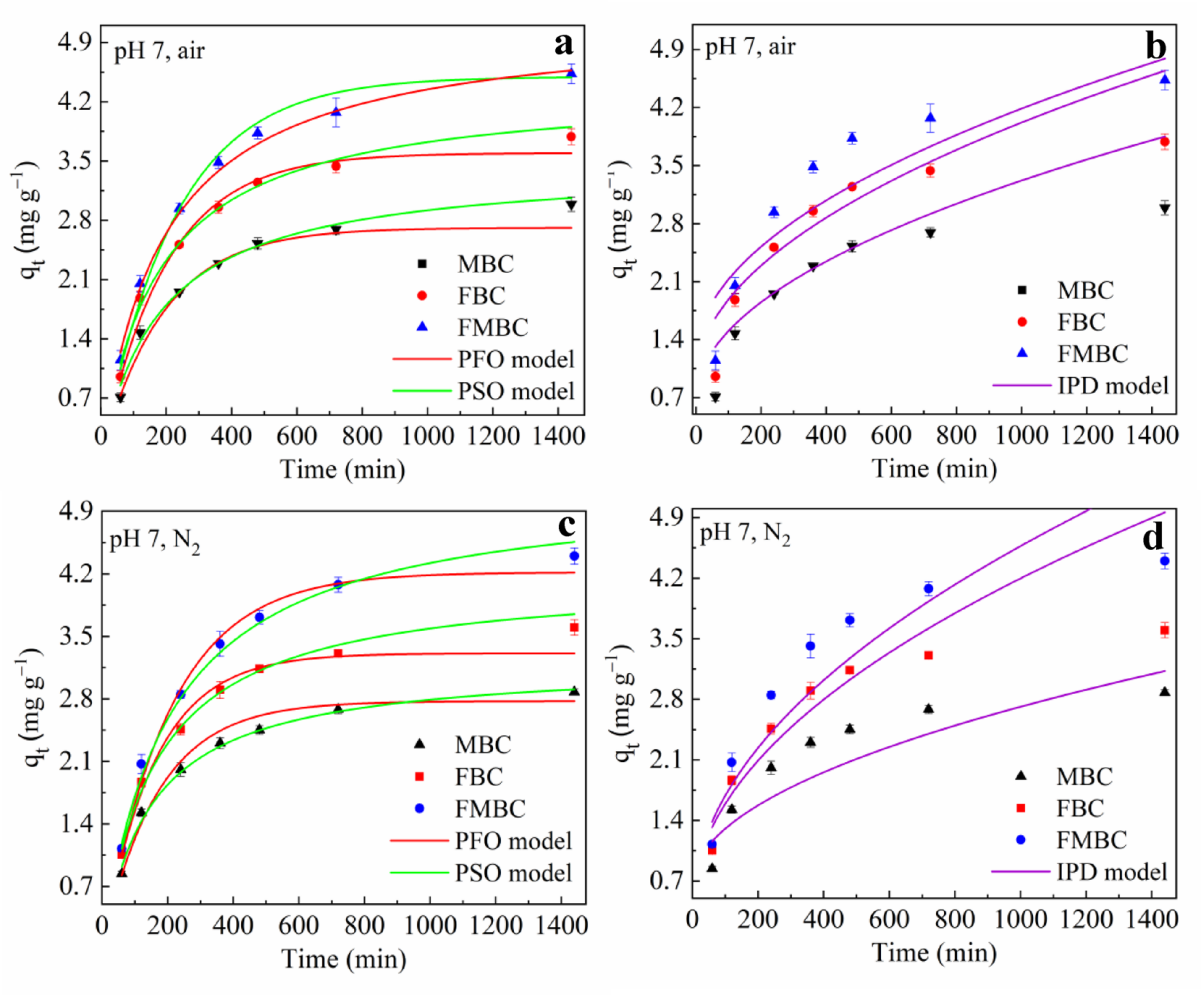
Kinetics fitting of the As(III) adsorption by MBC, FBC and FMBC at pH 7.0, in the presence of nitrogen (**a** and b) and air (**c** and **d**) atmospheres within 1440 min.

The As(III) adsorption kinetics of modified biochars (MBC, FBC, and FMBC) were evaluated under different atmospheric conditions using PFO, PSO, and IPD kinetic models (Table S1). As shown in Fig. 5, The PSO model provided a better fit than the PFO model, with calculated adsorption capacities (3.05, 3.88, and 4.48 mg g⁻¹ for MBC, FBC, and FMBC, respectively) closely matching the experimental values. The As(III) adsorption on MBCs occurred in two distinct stages: a rapid initial phase within the first 300 min, attributed to physisorption on readily accessible surface sites, followed by a slower approach to equilibrium^26^. Slow sorption might be due to irreversible chemisorption or the inner layer complex’s development^27^. Overall, The higher As(III) adsorption capacity of FMBC compared with MBC and FBC suggested that greater accessibility and availability of active adsorption sites in aqueous solution. The IPD model further confirmed that intraparticle diffusion played a crucial role in controlling the adsorption process^28^. Since the intercepts of fitting curves were not equal to zero, the removal of As(III) by modified BCs was not solely dependent on IPD^29^. The experimental data fit FMBC well (R^2^ > 0.9) based on the relationship between the adsorbed As (*q*_*t*_) and the time (*t*). Hence, As adsorption in air environments is significantly affected by IPD. These findings indicate that As(III) adsorption involved multiple steps, including solute diffusion, external mass transfer, and surface chemical reactions.

The results further reveal that Fe oxides primarily governed the adsorption process, whereas Mn oxides predominantly facilitated As(III) oxidation while exhibiting relatively low adsorption capacity across all mBCs.

Figures. S3 and S4 show the As(III) oxidation and adsorption by modified BCs at pH 7.0. The adsorption capacity of As noticeably increased in the first 240 min, and then the reaction rate gradually slowed until it reached equilibrium. As(III) adsorption and oxidation increased with the DO concentration. In MBC, FBC and FMBC systems at pH 7.0, the dissolved As(III) concentrations decreased to 12.09, 11.51 and 10.60 mg L^− 1^ in nitrogen atmosphere, and 11.85, 11.21 and 10.44 mg L^− 1^ in air atmosphere. The As(III, V) adsorption capabilities in nitrogen atmosphere were 2.87, 3.59, and 4.39 mg g^− 1^, and were 2.99, 3.78 and 4.60 mg g^− 1^ in air atmosphere. The oxidation of As(III) was evident in both MBC and FMBC systems, with the maximum As(V) concentrations detected at 120 min. Under anoxic conditions, As(V) reached 0.10 and 0.16 mg L^− 1^, whereas under oxic conditions, it increased to 0.15 and 0.20 mg L^− 1^ for MBC and FMBC, respectively, indicating enhanced oxidative transformation in the presence of dissolved oxygen. Then, no dissolved As(V) was detected at 1440 min. Similarly, the released Mn(II) was also increased to 0.12 and 0.10 mg L^− 1^ in nitrogen atmosphere and 0.10 and 0.07 mg L^− 1^ within 120 min in air atmosphere. Then, it was reduced until the concentrations were negligible in the solution after 1440 min (Fig. S4(c)). In the FBC system, no dissolved As(V) or Fe(II) was detected under the nitrogen atmosphere. In contrast, under oxic conditions, the dissolved As(V) concentration reached 0.08 mg L^− 1^ and subsequently declined to undetectable levels after 1440 min (Fig. S4(b)), indicating that Fe oxides served as the primary active phase governing the adsorption process in the presence of dissolved oxygen^14,30,31^. The above results indicated that the adsorption capacity of As by the modified BCs was improved after modification. The oxidation of As(III) enhanced As adsorption under different conditions.

During1440 minutes, OH^•^ was measured in the reaction systems at pH 7.0 in an air environment in the aqueous system that included Fe and Mn oxides loaded BC. The concentrations of OH^•^ were 1.35, 0.97 and 1.93 µM for MBC, FBC and FMBC, respectively. FMBC had the highest concentration of OH^•^ compared with FBC and MBC, which might accelerate As(III) oxidation. During the first 240 min, the cumulative concentration of OH^•^ displayed a remarkable increase, followed by a slow increase, then reached equilibrium in all the modified BCs (Fig. S5). Fe-Mn oxide enhanced As(III) adsorption and oxidation in the presence of DO.

### Study of pH, coexisting anions and regeneration efficiency

**Fig. 6:**
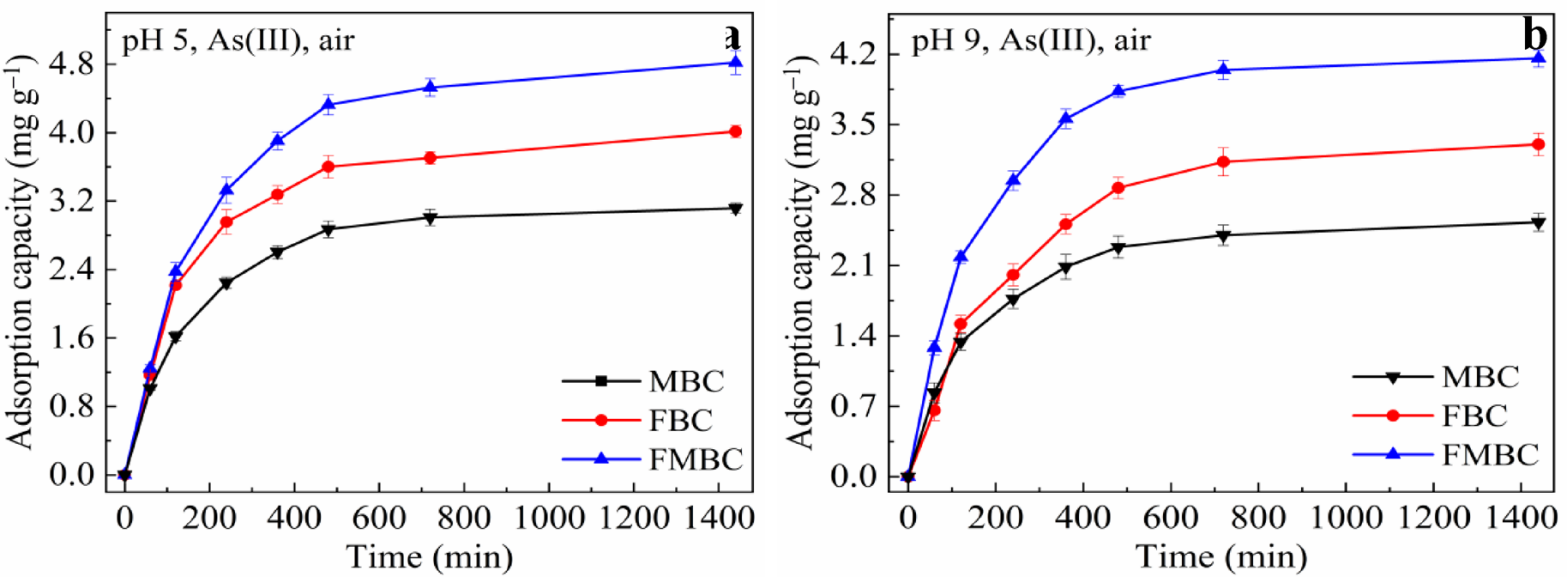
Adsorption capacities of As(III) by modified BCs at pH 5.0 (**a**) and pH 9.0 (**b**) under.air atmosphere within 1440 min.

Solution pH is a critical parameter that strongly influences both the adsorption behavior and oxidation efficiency of contaminants. The influence of pH values on As(III) adsorption and oxidation on modified BCs is shown in Fig. 6 and Table S2. The As adsorption capacities of MBC, FBC and FMBC at pH 5.0 were 3.23, 4.01 and 4.85 mg g^− 1^, respectively. When the pH was increased to 9.0, the corresponding values declined to 2.52, 3.30 and 4.17 mg g^− 1^, respectively. The initial pH slightly affected As(III) adsorption performance on FMBC. However, the As adsorption on MBC and FMB was highly affected by pH. They showed excellent As adsorption and oxidation abilities at pH 5.0. By increasing the pH, their adsorption efficiency decreased. The pH value is generally considered an essential factor affecting oxidation performance. In MBC, FBC and FMBC systems, the adsorbed As(V) were 1.55, 1.28 and 3.24 mg g^− 1^ at pH 5.0 (Table S2). There was a decrease in the values corresponding to 0.88, 0.72 and 2.05 mg g^− 1^ when pH was increased to 9.0. The increase in adsorbed As(V) at pH 5.0 indicated that the As(III) oxidation by modified BCs was pH-dependent. FMBC notably oxidized As(III) to As(V) under different conditions.

Based on these results, modified BCs enhanced As(III) removal by oxidizing As(III) to As(V), with the latter potentially re-adsorbed on their surfaces. The above results suggested that FMBC was more appropriate for removing As in a wide pH range.

The effects of four common coexisting anions (PO₄^3−^, SiO₃^2−^, HCO₃^3−^, and SO₄^2−^) on the adsorption efficiency of As(III) were systematically investigated (Fig. S6). PO_4_^3−^ had a substantially greater impact on As(III) removal efficiency than the other anions. In the presence of PO₄^3−^, the As(III) removal efficiency decreased to 33.72%, 43.84%, and 53.93% for MBC, FBC, and FMBC, respectively, indicating that As(III) adsorption in the FMBC system was least influenced by competing anions. The desorption and cycling capacity tests were conducted using four adsorption-desorption cycles, as shown in Fig. S7. In the first cycle, the adsorption capacities for As(III) were 2.93, 3.96 and 5.19 mg g^− 1^ for MBC, FBC and FMBC, while in the fourth cycle, these capacities had decreased to 1.54, 2.41 and 3.82 mg g^− 1^ for MBC, FBC and FMBC, respectively. In brief, the adsorption–desorption results showed that FMBC has a much higher cycle capacity than MBC and FBC, and demonstrated consistent performance over several use cycles.

## Discussion

### As(III) adsorption and oxidation mechanisms

As typically exists in anionic species under environmental conditions; therefore, its interaction with carboxylic and phenolic functional groups on biochar surfaces is often limited due to electrostatic repulsion and low surface affinity^32^. Investigating the changes in surface functional groups following the reaction using FTIR analysis is essential to better comprehending the process of As adsorption and oxidation on MBCs (Fig. 1). Following As adsorption, the bands at 1695, 1589, and 802 cm^− 1^ shifted slightly to 1700, 1593, and 808 cm^− 1^, respectively, while the bands at 2915 and 2861 cm⁻¹ became more intense and well-defined, indicating changes in surface functional group interactions. (Fig. 1(d))^18^. The results indicated that − OH groups act as key sorption sites, facilitating As(III) and As(V) adsorption on modified biochars through surface complexation and ion-exchange mechanisms^7^. In addition, As adsorption from aqueous solutions was also enhanced by = CH_2_ − CH_3_ and − COOH surface functional groups. These findings coincided with earlier studies, which reported that the − OH and − COOH groups were responsible for As(V) removal, whereas As(III) was mainly bound to C − O, =CH_2_/−CH_3_ and at least partially to − OH^2,27^. In the As(V) range, As was adsorbed on the material’s surface between 650 and 950 cm^− 1^ as As − O bands, indicating that the As(III) oxidation occurred on all modified BCs surfaces^33^.

To further clarify, As adsorption and oxidation on Fe-Mn modified BCs, XPS analysis were required to investigate the valence state and role of Fe and Mn oxides. Fig. S8 shows the Mn 2p distinctive peaks’ XPS spectra and quantitative fitting results before and after As adsorption. For MBC systems, the binding energies of Mn 2p shifted from 641.53 to 645.05 to 641.75 and 645.47 eV in nitrogen atmosphere and 642.00 and 645.93 eV in air atmosphere (Figs. S8(a-c)). In the FMBC system, the binding energies changed from 642.14 to 645.87 to 642.20 and 646.10 eV in nitrogen atmosphere and 642.18 and 645.80 eV in air atmosphere (Figs. S8(d-f)). The relative proportion and composition of Mn oxides in the MBC spectrum remained unchanged following As(III) adsorption, indicating their structural stability during the reaction. Under nitrogen and air atmospheres, the proportion of Mn(IV) in MBC increased from 55.71% to 86.01% and 57.20%, respectively, while Mn(III/II) decreased from 44.99% to 13.99% and 24.80%. In contrast, for the FMBC system, the Mn(IV) content decreased from 42.28% to 27.38% under nitrogen and 39.37% under air, accompanied by an increase in Mn(III/II) from 57.71% to 72.62% and 60.63%, respectively. It was found that a redox reaction occurred during the adsorption process. The result as illustrated in Fig. S4 may be supported by the liberated Mn(II) and dissolved As(V).

Mn(IV) plays a crucial role in the redox process, initially adsorbing dissolved As(III) and subsequently oxidizing it to As(V), during which Mn(IV) is partially reduced to Mn(III/II). The relative content of the Mn(III/II) peak of MBC and FMBC after reaction with As(III) was higher than that of the pristine MBC and FMBC. This was due to the precipitation of Mn(II) and As(III/V) or re-adsorption of Mn(II) on adsorbent surfaces^21^. Based on the previous mechanism, the Mn oxide transformations were affected by DO concentration in MBC and FMBC samples. As(III) oxidation activity was more significant in FMBC than in MBC. Moreover, Mn oxide content slightly changed during the reaction.

The quantitative fitting findings of the Fe 2p characteristic peaks prior to and following the As adsorption are displayed in Fig S9. Fe 2p_3/2_ and Fe 2p_1/2_ were assigned two strong asymmetric peaks at 711.01 eV and 724.72 eV, respectively. Two separate forms of Fe(III) were linked to binding energies of 711.82 eV and 713.21 eV. Additionally, the existence of Fe(III) was determined by the shake-up satellite peak at around 718.62 eV. After adsorption, there was no apparent change in the structure (Figs. S9(b, c, e and f)) since Fe atoms were located in the structure’s core and could not be affected by As adsorption under different atmospheres. For the FMBC spectrum, the percentage of Fe_2_O_3_ decreased from 55.01 to 48.66 and 48.07% and that of FeOOH increased from 26.85 to 27.28 and 27.52% in nitrogen and air atmospheres, respectively. While the relative content of Fe_2_O_3_ in the FBC system decreased from 44.47 to 41.84 and 39.76%, and that of FeOOH increased from 28.63 to 30.92 and 33.86% in nitrogen and air atmospheres, respectively, showing strong interactions between As(III) and FeOOH and indicating that As adsorption was primarily caused by Fe oxides. As(III) and iron oxides underwent a somewhat sluggish redox reaction, and the solution did not include any dissolved Fe(II) ions during the process^1^. Several studies have shown that FeOOH could easily co-precipitate with As, which enhanced its removal^34^. According to the results, As adsorption increased in the air more than in the nitrogen atmosphere, suggesting that the oxidation process improved As adsorption.

**Fig. 7:**
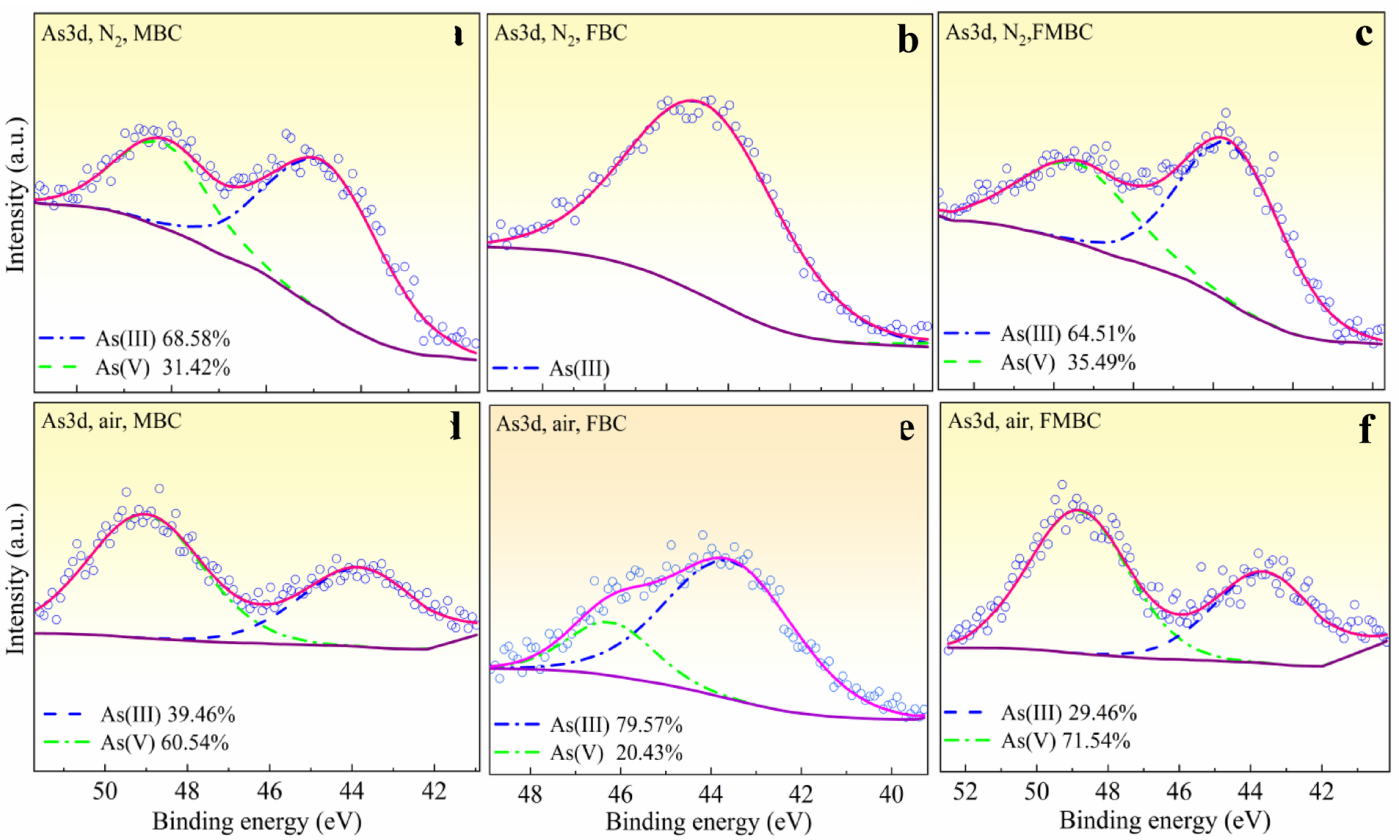
XPS As 3 d spectra for MBC, FBC and FMBC systems before and after As(III) adsorption at pH7.0.

Figure 7 shows the As 3 d spectra in modified BCs. The As 3 d peaks at 43.91 eV and 46.70 eV, corresponding to As(III) and As(V), respectively, indicate that oxidative co-adsorption of As(III) on the adsorbents led to the effective immobilization of arsenic species on their surfaces. According to the results, As(III) oxidation on MBC and FMBC was accelerated under oxic conditions. Accordingly, As(III) was effectively oxidized to As(V), with the latter adsorbed on the MBC and FMBC surfaces. However, the single peak for FBC in the air environment was found at 45.83 eV, which corresponds to As(V).

In the absence of dissolved oxygen, no significant oxidation of As(III) was observed (Figs. 7b and e), indicating that the redox interaction between As(III) and Fe oxides proceeded at a relatively slow rate^15,35^.

### Catalytic oxidation of As(III)

As(III) was adsorbed in various atmospheres by MBC, FBC, and FMBC in the current investigation; the oxidation of As(III) likely enhanced the overall adsorption capacity, particularly under oxic conditions, where redox reactions and adsorption processes occurred concurrently. MnO_2_ was primarily responsible for the As(III) oxidation process in MBC and FMBC systems as shown in Eqs. (4) and (3)^21^. The adsorbed As(III) on the Mn-oxide surface via the –OH groups in the form of bidentate inner-sphere complexes caused the oxidation of As(III) to As(V) and the reduction of Mn(IV) to Mn(III) and Mn(II). A portion of As(V) could be adsorbed on FMBC and MBC when As(V) and Mn(II) were desorbed into the solution (Eq. 4). In some cases, the dissolved As(V) and released Mn(II) could react together, precipitating As(V) with Mn(II) (Eq. 3)^35,36^.

2 $${\mathrm{2Mn}}-{\mathrm{O}}{{\mathrm{H}}_{({\mathrm{s}})}}\,+\,{{\mathrm{H}}_{\mathrm{3}}}{\mathrm{A}}{{\mathrm{s}}^{\mathrm{V}}}{{\mathrm{O}}_{{\mathrm{4}}({\mathrm{aq}})}} \to {\left( {{\mathrm{MnO}}} \right)_{\mathrm{2}}}{\mathrm{A}}{{\mathrm{s}}^{\mathrm{V}}}{\mathrm{OO}}{{\mathrm{H}}_{({\mathrm{s}})}}\,+\,{\mathrm{2}}{{\mathrm{H}}_{\mathrm{2}}}{\mathrm{O}}$$

3 $${\mathrm{Mn}}{\left( {{\mathrm{II}}} \right)_{({\mathrm{aq}})}}\,+\,{{\mathrm{H}}_{\mathrm{2}}}{\mathrm{A}}{{\mathrm{s}}^{\mathrm{V}}}{{\mathrm{O}}_{\mathrm{4}}}{^{ - }_{({\mathrm{aq}})}}\,+\,{{\mathrm{H}}_{\mathrm{2}}}{\text{O }} \to {\mathrm{MnHA}}{{\mathrm{s}}^{\mathrm{V}}}{{\mathrm{O}}_{\mathrm{4}}}.{{\mathrm{H}}_{\mathrm{2}}}{\mathrm{O}}\left( {\mathrm{s}} \right)\,+\,{{\mathrm{H}}^+}$$

Re-adsorption on modified BCs with negatively charged surfaces, precipitation with As(V), or oxidation under the catalytic action of Mn-oxide in the presence of DO cause a decrease in Mn(II) concentration^1^. There were no changes in the Fe content of FMBC and FBC, indicating that Fe oxides do not cause As (III) oxidation. On Fe oxides, As(V) becomes more strongly adsorbed than As(III) at most typical pH values. Lewis acid-base and Coulombic (electrostatic) interactions are necessary to explain the adsorption of As(V), while Fe oxide may adsorb As(III) according to Lewis acid-base interactions. As (III) and As (V) surface complexation are more likely to occur when FeOOH is used as an adsorbent (Eq. 4)^37^. The adsorption of As onto the FeOOH surface occurs through − OH groups via ligand exchange, leading to the formation of monodentate, bidentate, or binuclear bridging complexes at relatively lower adsorption rates^37,38^.

4 $${\mathrm{Fe}}{\left( {{\mathrm{OH}}} \right)_{\mathrm{3}}}+{\text{ }}{{\mathrm{H}}_{\mathrm{3}}}{\mathrm{As}}{{\mathrm{O}}_{\mathrm{4}}} \to {\text{ FeAs}}{{\mathrm{O}}_{\mathrm{4}}}\cdot{\mathrm{2}}{{\mathrm{H}}_{\mathrm{2}}}{\mathrm{O}}\,+\,{{\mathrm{H}}_{\mathrm{2}}}{\mathrm{O}}$$

As(III) adsorption and oxidation were improved after modification in the presence of DO. As(III) was adsorbed on the Mn-oxide surface of FMBCs and MBCs, resulting in reduction of Mn(IV) to Mn(III) and Mn(II) and oxidation of As(III) to As(V). Furthermore, it was observed that there was no oxidation occurring in the FBC system under nitrogen conditions for 1440 min (Fig. 7(b)). As (III) and As (V) surface complexation are more likely to occur when FeOOH is used as an adsorbent. As(III) oxidation in FBC was probably caused by redox-active moieties derived from biochar. The modified BCs were the best method in As adsorption and oxidation. Moreover, FMBC was the most efficient material for As adsorption capacity under air atmosphere.

The catalytic activity of solid catalysts is determined by their surface charge properties. Due to its multivalence oxidation, MnO_x_ has high catalytic activity^39,40^. Mn-oxides may oxidize As(III) without the need for DO, according to the results of As(III) oxidation in air and nitrogen atmospheres. Additionally, FMBC exhibited excellent As adsorption via the same redox mechanism, as previously described by Lin, et al.^14^. Moreover, it’s possible that the oxidation of As(III) was not dependent on direct oxygen because of the production of OH^•^ (Fig. S5). Recently, BC has provided electron-accepting moieties such as quinones, especially for thermal BC pyrolyzed at high temperatures, which are likely responsible for the As oxidation. In contrast, Fe-oxides could catalyze As(III) oxidation in the presence of DO^31^. As(III) oxidation in FBC was likely caused by redox-active moieties derived from the biochar. Klüpfel et al.^41^, reported that pyrolyzed biochar at high temperatures could provide electron-accepting quinones. Additionally, redox-active moieties were likely responsible for As(III) oxidation in the presence of DO. Consequently, the presence of oxygen explained the considerable increase in the production of OH^•^ during the reaction, which facilitated the oxidation of As (III). Besides, the OH^•^ generated from electron-donating moieties might oxidize As(III) on FBC^42,43^. Fe(III) has a high redox potential, so persistent free radical formation rates are highest when it is present^44^.

### Solution pH, co-anion and reusability efficiency

The adsorption and oxidation of As by modified BCs are critically dependent on the pH and primarily rely on the primary form of As in the solution as well as the surface charge of modified BCs. The PZC of iron oxides is about 7.0–9.0, and that of manganese oxides is about 2.0–3.0^2,45^. In this study, Because the adsorption was carried out at a pH of 5.0 to 9.0, MBC’s surface charge was negative, which restricted its ability to adsorb As(III, V). On the other hand, both FBC and FMBC have positive charge until pH = 6.5. The pH of the solution had no effect on the removal of As(III); instead, a little decrease in adsorption was seen as the pH of the solution changed (Fig. 6). Adsorption efficiency could be improved when the As anion and the adsorbents had opposite charges during the adsorption. Fe-Mn alteration of biochar boosts its positive surface charge, according to zeta potential measurements (Fig. S2). This positive charge enhances the attraction of negatively charged arsenic species (especially arsenite and arsenate) through electrostatic interactions, making the biochar more effective for As adsorption. Acidic conditions led to higher positive charges on the material’s surface and greater protonation ability, improving As adsorption. In alkaline conditions, the OH^−^ accumulated and enhanced the surface’s negative charges, resulting in repulsion between modified BCs and As anions, which reduced As adsorption capacity^46^. Therefore, creating a stronger repulsive force against the As anion when the solution’s pH was higher than PZC. Furthermore, Anionic forms of As(V) have always existed, including H_2_AsO_4_^–^ (pH < 7) and HAsO_4_^2–^ (pH > 7)^47–49^. According to this characteristic, when the pH increased, a negative charge on the samples’ surfaces would repel As(V) ions more electrostatically, reducing their ability to remove As(V). Conversely, As(III) was dominated by nonionic H_3_AsO_3_ species (pH < 9.1) and anionic H_2_AsO_3_^−^ species (pH > 9.1). Therefore, As(V) has a more negative charge; thus, adsorbed As(V) was lower than adsorbed As(III) under alkaline conditions^21^.

PO_4_^3−^dramatically reduced the removal efficiency of As(III) in comparison to other anions. Because of weak acid dissociation, the adsorbent surface’s OH and negative charge increased. PO_4_^3−^ may change the chemical equilibrium, solution’s conductivity and, pH which might lead to changes in ion diffusion rates and less As adsorption^50^. Due to their similar exterior electron configurations and group membership, PO_4_^3−^ and As-oxyanion may compete with As for adsorption sites. Additionally, both are adsorbed onto iron oxides as an inner-sphere complex. Both arsenate and PO_4_^3−^ are oxyanions in aqueous solution that have three analogous, pH-dependent species. PO_4_^3−^ and As(V) can compete even though As(V) adsorbs on iron (hydr)oxides more strongly. The pH of the solution, anion concentrations, As(V) loading on the adsorbent surface, the kind of bonding, and the adsorbent’s surface characteristics all affect both direct and indirect effects. The reduction in the number of accessible surface sites may be one reason why PO_4_^3−^ competition increases with higher PO_4_^3−^concentrations. Competition is absent if the adsorbent’s capacity is enough for the adhesion of both ions^51,52^. As adsorption performance was less affected by coexisting ions in the FMBC system.

After each adsorption–desorption cycle, a residual adsorption capacity may persist, likely due to the generation of new active sites and the partial consumption or reorganization of surface functional groups during structural regeneration. Furthermore, after the initial cycle, the adsorption capacity of every adsorbent for As(III) consistently dropped; this decline in adsorption efficiency might be linked to a decrease in specific surface area and the weakening of functional groups on all adsorbents following repeated usage^53^. To examine the regeneration performance and enhance the number of cycles, future research will need to employ various desorbents (such KNO_3_, NaCl, and EDTA). Therefore, in future studies, other valence metals can be taken into consideration. Additionally, using the treated wastewater solution to irrigate farming soil in order to raise the mineral content is a potential tactic.

According to earlier findings, all modifications have enhanced the BC properties for As oxidation and adsorption. Finally, the FMBC was the best alternative for As(III) adsorption and oxidation when compared with FBC and MBC. Overall, the functional groups on the BC and Fe-Mn oxide particles could directly adsorb As(III) and As(V) via the surface complexation mechanism and contributed to the improvement of adsorption efficiency (Fig. S10).

## Conclusion

The modification of BC significantly improved its capacity for As(III) adsorption and oxidation. As(III) is adsorbed by modified BCs at varying temperatures in accordance with the PSO and Langmuir-Freundlich models, and adsorption is enhanced by suitably raising the temperature. Among the modified biochars, FMBC demonstrated the highest efficiency due to the synergistic effects of Mn-driven oxidation and Fe-facilitated adsorption. The adsorption process was influenced by pH, coexisting anions, and DO, with FMBC showing consistent performance under varied conditions. These findings indicate that FMBC is a highly effective, low-cost, and environmentally friendly material for the remediation of arsenic-contaminated water systems. Future research should focus on improving this approach and evaluating its long-term stability, reusability, economic sustainability, regeneration effectiveness, and toxicity testing. Real-world applications of Fe-Mn modified biochar (FMBC) in wastewater and soil remediation.

## Supplementary Information

Below is the link to the electronic supplementary material.

Supplementary Material 1

## Data Availability

All data generated or analyzed during this investigation are included in this published article.
