# Supplementary material for: Synergistic adsorption and oxidation of arsenite by Fe–Mn binary oxide-modified bamboo biochar in the presence of air
Source: Sci Rep. 2025 Dec 27;15:44650. doi: 10.1038/s41598-025-32178-5 (PMC12749615; doi:10.1038/s41598-025-32178-5)
Supplement: Supplementary file 1 — Supplementary Material 1 [file 41598_2025_32178_MOESM1_ESM.docx]

**Supplementary Materials for**

**Synergistic adsorption and oxidation of arsenite by Fe–Mn binary oxide-modified Bamboo biochar**

**Omar Rady ^1,^*, Ahmed Bakr^2^, Mohamed G Moussa^3^, Belal Nodhy^4^**

1. Soils and water department, Faculty of Agriculture, Al-Azhar University, Cairo 11651, Egypt.
2. Environment and Bio-agriculture Department, Faculty of Agriculture, Al-Azhar University, Cairo 11651, Egypt.
3. Soil and Water Research Department, Nuclear Research Center, Egyptian Atomic Energy Authority, Cairo 13759, Egypt.
4. Central Laboratory for Environmental Quality Monitoring (CLEQM), National Water Research Center (NWRC), Egypt.

* **Corresponding Author:** Omar Mohamed Rady Abdellateef

Email: [omarrady82@azhar.edu.eg](mailto:omarrady82@azhar.edu.eg)

**Methods**

After adsorption, the crystal structure of the adsorbents was characterized by powder X-ray diffraction (Philips PW3710, diffractometer), with Cu Kα radiation, scanning 2θ from 10° to 70° with a 2° min^−1^ rate. Fourier transform infrared spectroscopy (FTIR, Nicolet iS50, Thermo Fisher Scientific) was conducted with pellets made from KBr powders under manual hydraulic pressure to study the functional groups of samples. The surface chemical composition and the valence states of the solid products were characterized by XPS (PHI 5000 Versa Probe, USA) with Al Ka at 1486.71 eV. The charge effect was corrected by adjusting the binding energy (BE) of C 1s to 284.62 eV. The Shirley-type background was subtracted before deconvolution and fitting. The parameters used by Nesbitt et al. [1] for the multiplet peaks of Mn 2p_3/2_ for spectra fitting were adopted. A 20:80 ratio of the Lorentzian:Gaussian mix-sum function was used for all the fittings. An automatic analyzer (Quantachrome, USA) was applied to determine the total pore volume (V_tot_) and the specific surface areas of samples using Brunauer-Emmett-Teller (BET) technique. Point of zero charge (PZC) was estimated using Mastersizer 2000 (Malvern Co.). The material contents of C, N, H and S in the samples were detected using elemental analyzer (Flash 2000, Thermo Fisher Scientific). The concentrations of As were analyzed using hydride generation atomic fluorescence spectroscopy (AFS- 9760, China). The contents of C, N, H and S in the samples were detected using an elemental analyzer (Flash 2000, Thermo Fisher Scientific). The possibility of hydroxyl radical (OH^•^) formation in the reaction systems was investigated by high-performance liquid chromatography (HPLC, Agilent 1200).

**Tables**

**Table S1** The As(III) adsorption kinetics parameters by different modified BCs under different conditions.

| **Samples** | **PFO model** | | |  | **PSO model** | | |  | **IPD model** | | | |
| --- | --- | --- | --- | --- | --- | --- | --- | --- | --- | --- | --- | --- |
|  | *q_e_* | *k_1_ x 10^−2^* | *R^2^* |  | *q_e_* | *k_2_ x 10^−2^* | *R^2^* |  | *C* | *k_3_ x 10^−2^* | *R^2^* |  |
| **MBC, pH 7, N_2_** | 2.77 | 0.58 | 0.985 |  | 2.91 | 0.21 | 0.994 |  | 0.65 | 6.5 | 0.867 |  |
| **MBC, pH 7, air** | 2.71 | 0.52 | 0.986 |  | 3.05 | 0.15 | 0.995 |  | 0.66 | 8.4 | 0.836 |  |
| **FBC, pH 7, N_2_** | 3.31 | 0.62 | 0.996 |  | 3.73 | 0.14 | 0.995 |  | 0.38 | 12.07 | 0.812 |  |
| **FBC, pH 7, air** | 3.59 | 0.50 | 0.987 |  | 3.88 | 0.12 | 0.995 |  | 0.49 | 9.88 | 0.813 |  |
| **FMBC, pH 7, N_2_** | 4.21 | 0.48 | 0.995 |  | 4.52 | 0.09 | 0.996 |  | 0.13 | 13.5 | 0.881 |  |
| **FMBC, pH 7,air** | 4.55 | 0.44 | 0.992 |  | 4.48 | 0.10 | 0.997 |  | 0.17 | 9.53 | 0.882 |  |

**Table S2** As adsorption capacities by modified BCs under different pH.

| **Samples** | **Adsorption capacity (mg g^−1^)** | | |
| --- | --- | --- | --- |
|  | **Adsorbed**  **As(III)** | **Adsorbed**  **As(V)** | **Total**  **As** |
| **MBC, pH 5** | 1.68 | 1.55 | 3.23 |
| **FBC, pH 5** | 1.81 | 1.28 | 4.01 |
| **FMBC , pH 5** | 1.56 | 3.24 | 4.80 |
| **MBC, pH 7** | 1.83 | 1.14 | 2.97 |
| **FBC, pH 7** | 2.83 | 0.96 | 3.79 |
| **FMBC, pH 7** | 1.90 | 2.76 | 4.66 |
| **MBC, pH 9** | 1.64 | 0.88 | 2.52 |
| **FBC, pH 9** | 2.58 | 0.72 | 3.30 |
| **FMBC, pH 9** | 2.12 | 2.05 | 4.17 |

**Figures**


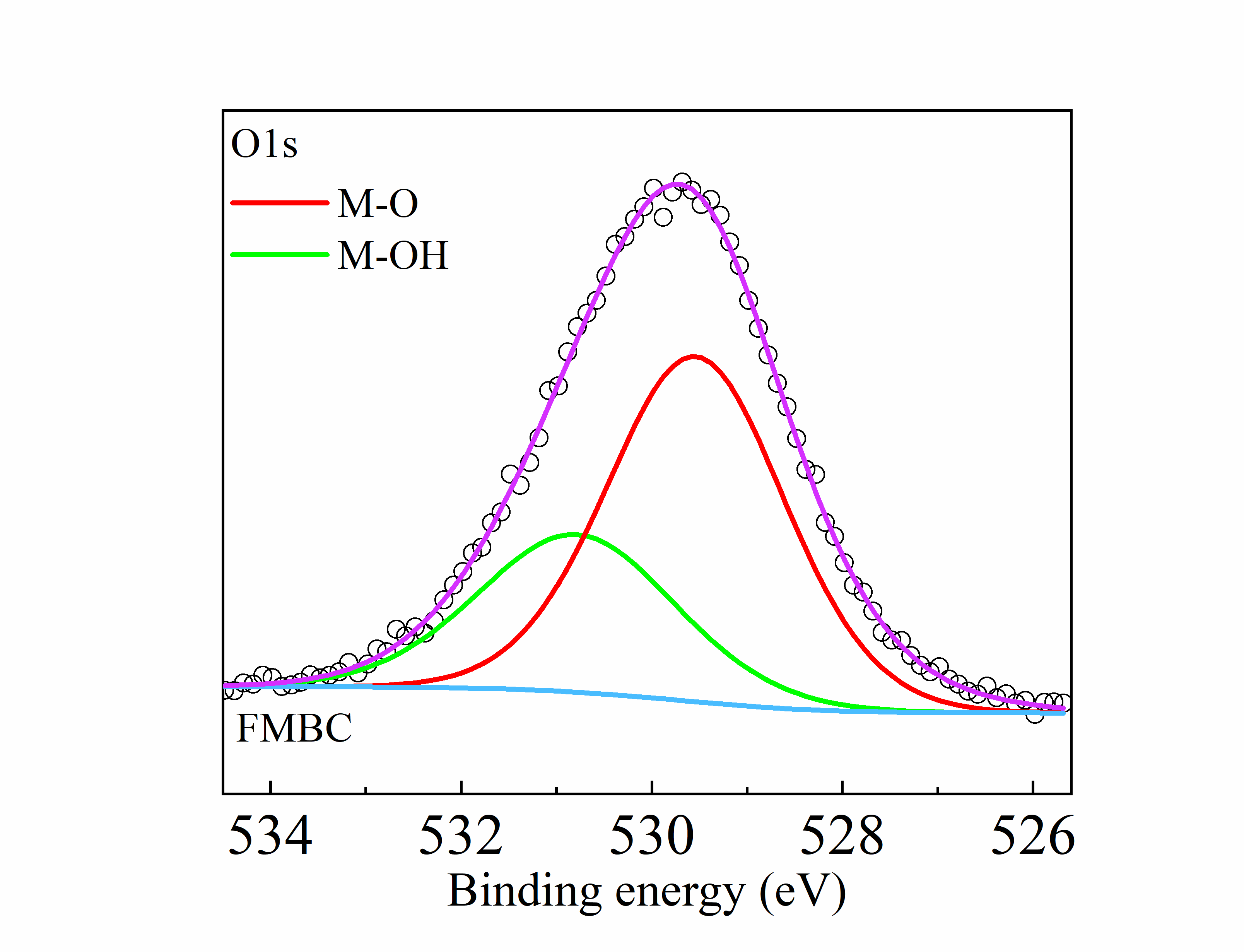

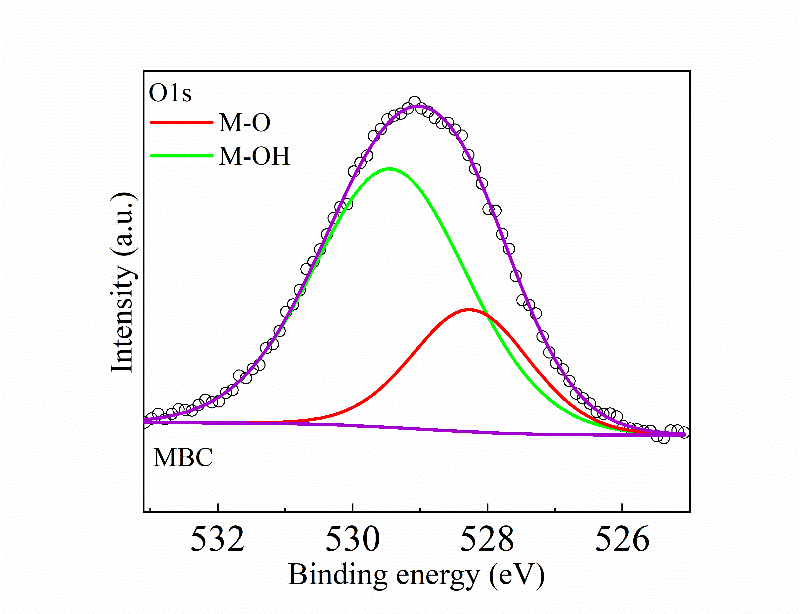

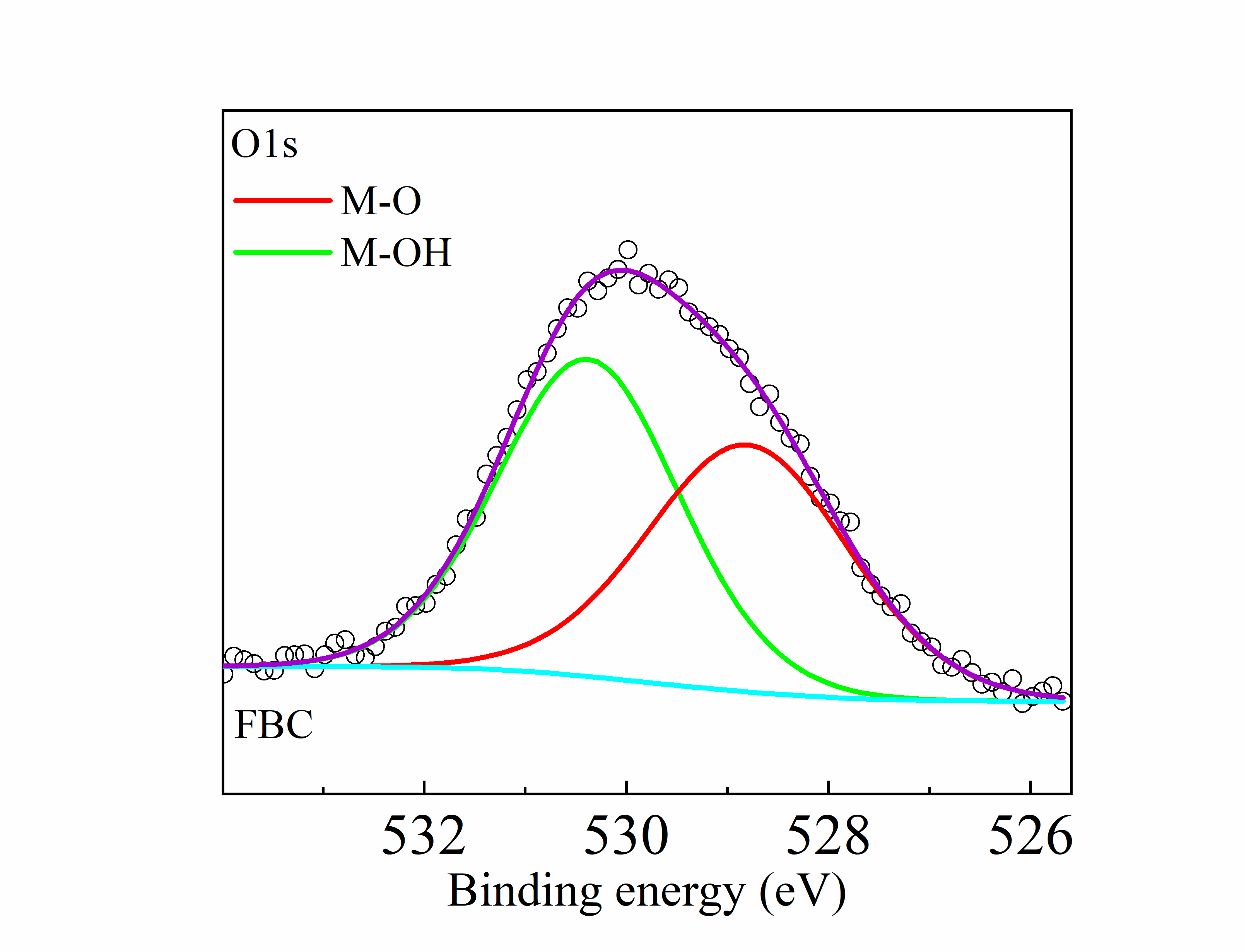

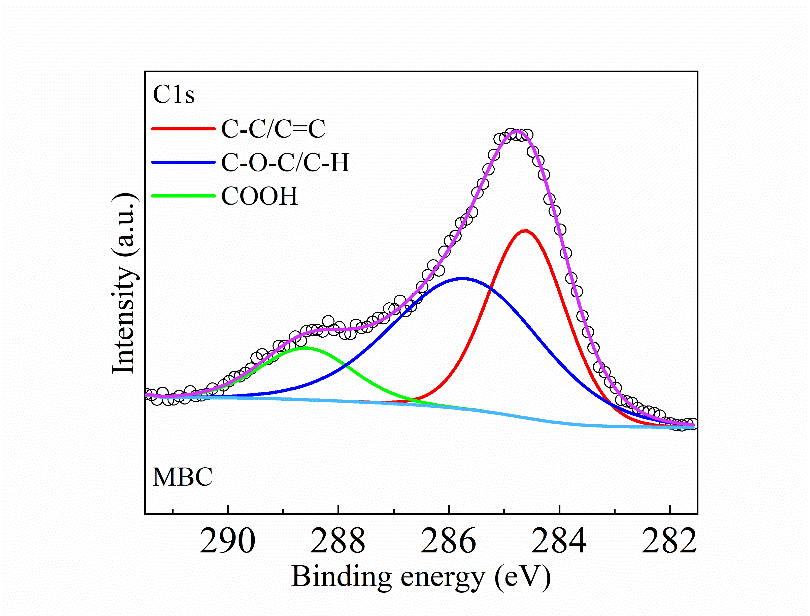

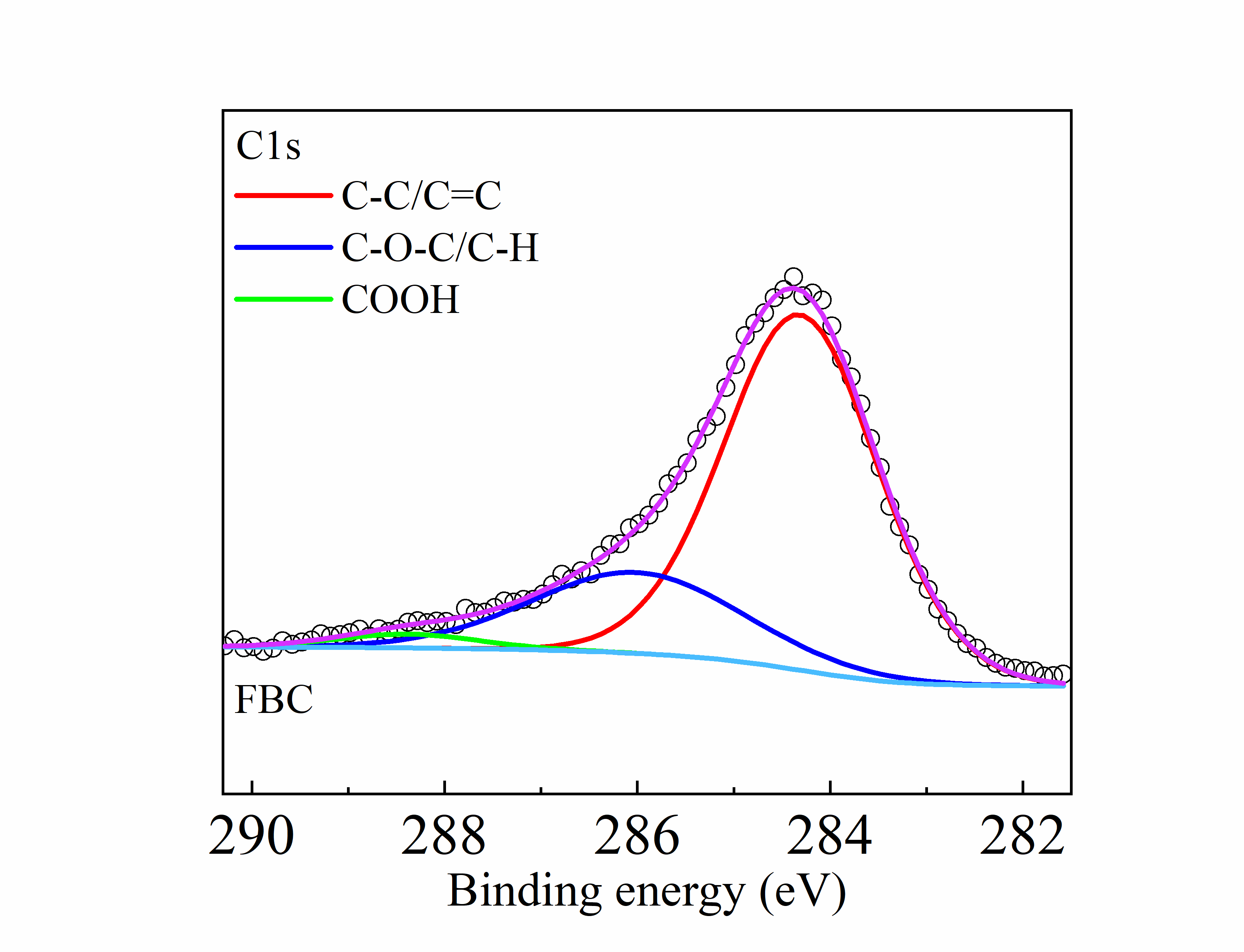

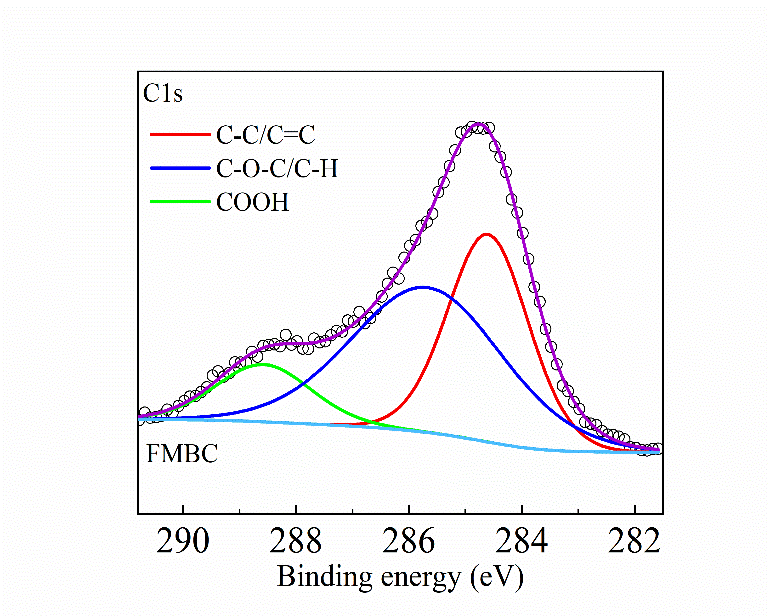


**f**

**b**

**a**

**c**

**d**

**e**

**Fig. S1** XPS O 1s (a, b and c) and C 1s (d, e and f) spectra of modified BCs (FBC, FMBC and MBC) before reaction





**Fig. S2** Zeta potential of all adsorbents measured at different pH.

**Fig. S3** Concentrations of dissolved and adsorbed As species in the reaction systems with 15 mg L^−1^ As(III) and 1.0 g L^−1^ of MBC (a and b), FBC (c and d) and FMBC (e and f ) at pH 7.0 within 1440 min in nitrogen and air atmospheres.


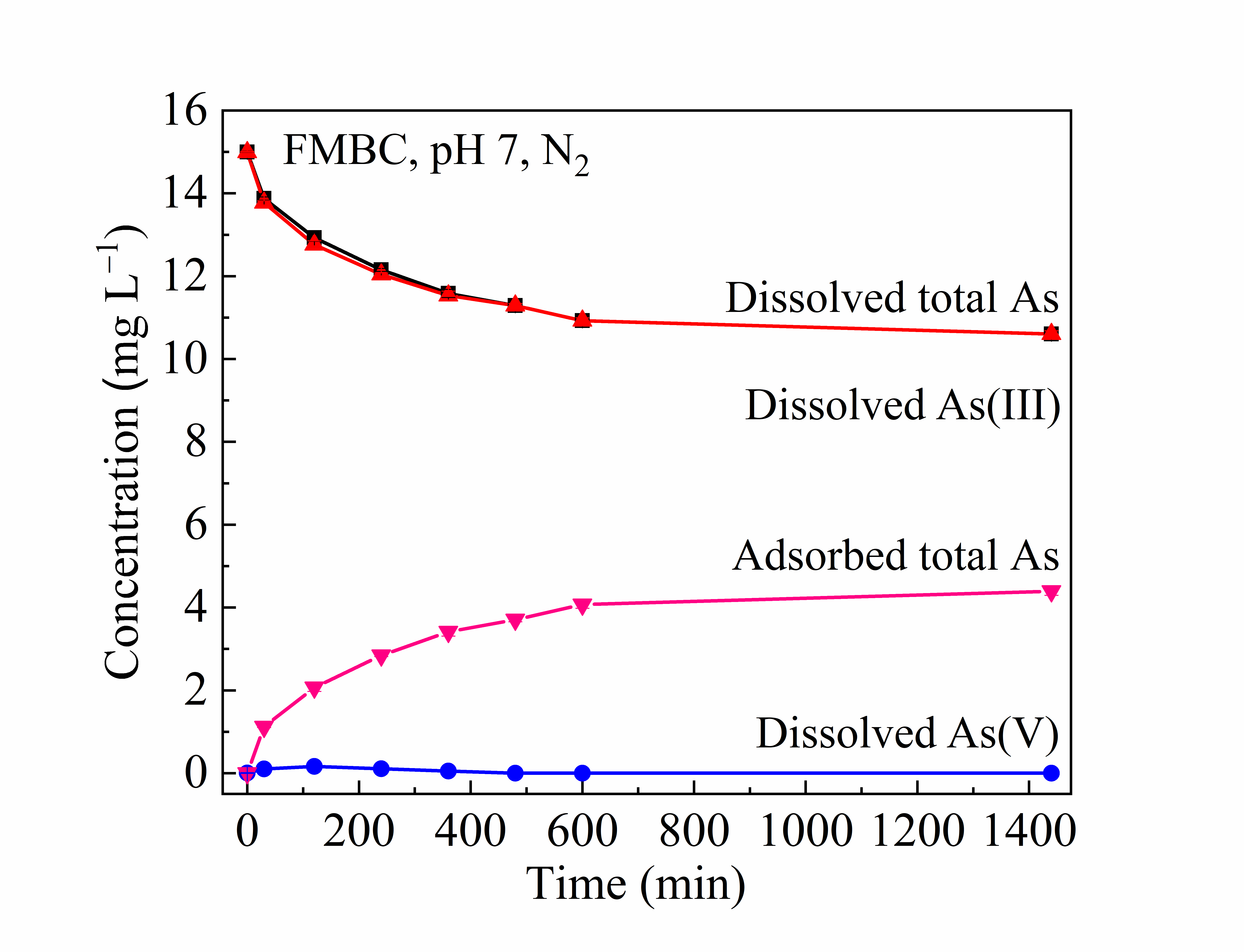


**c**


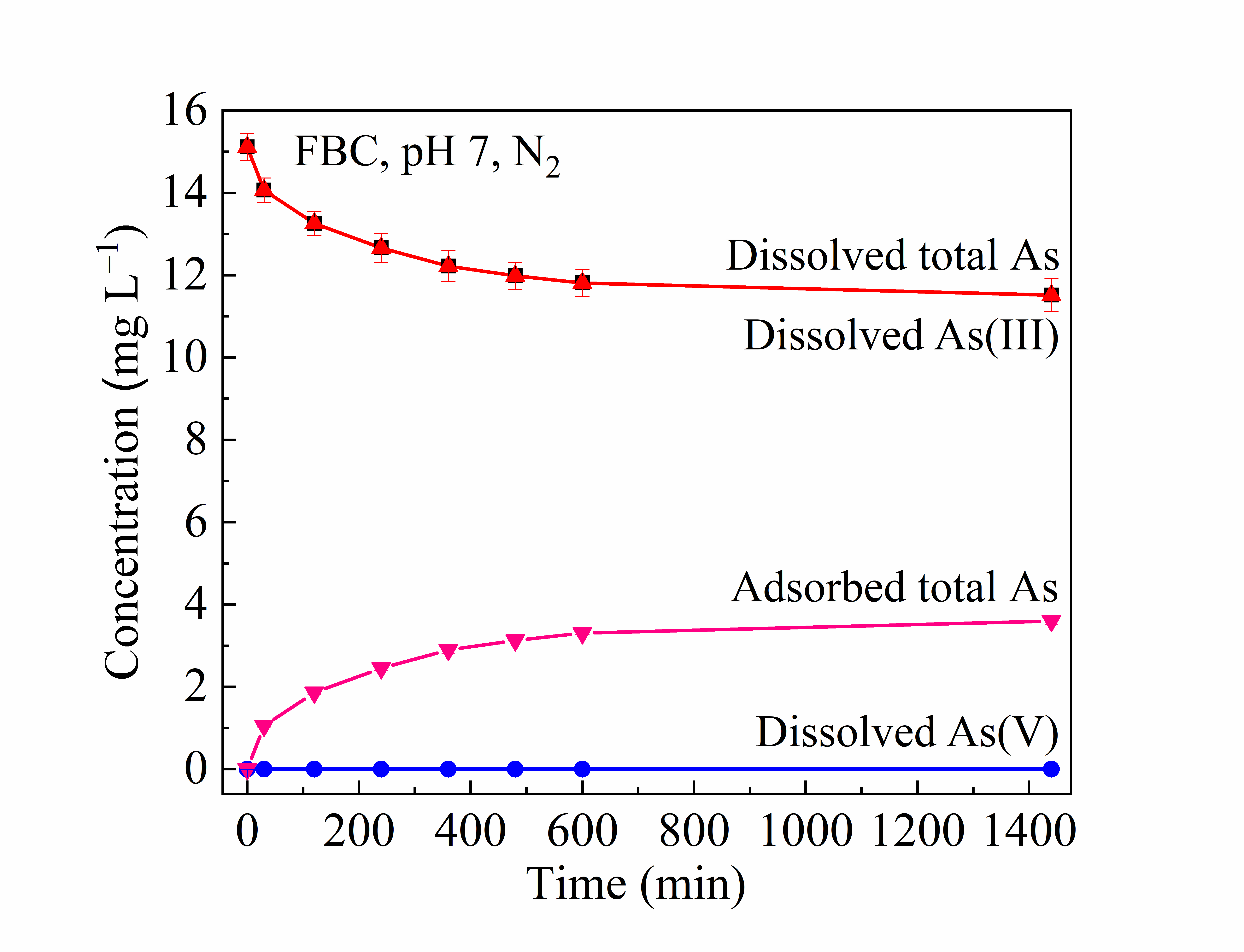


**b**


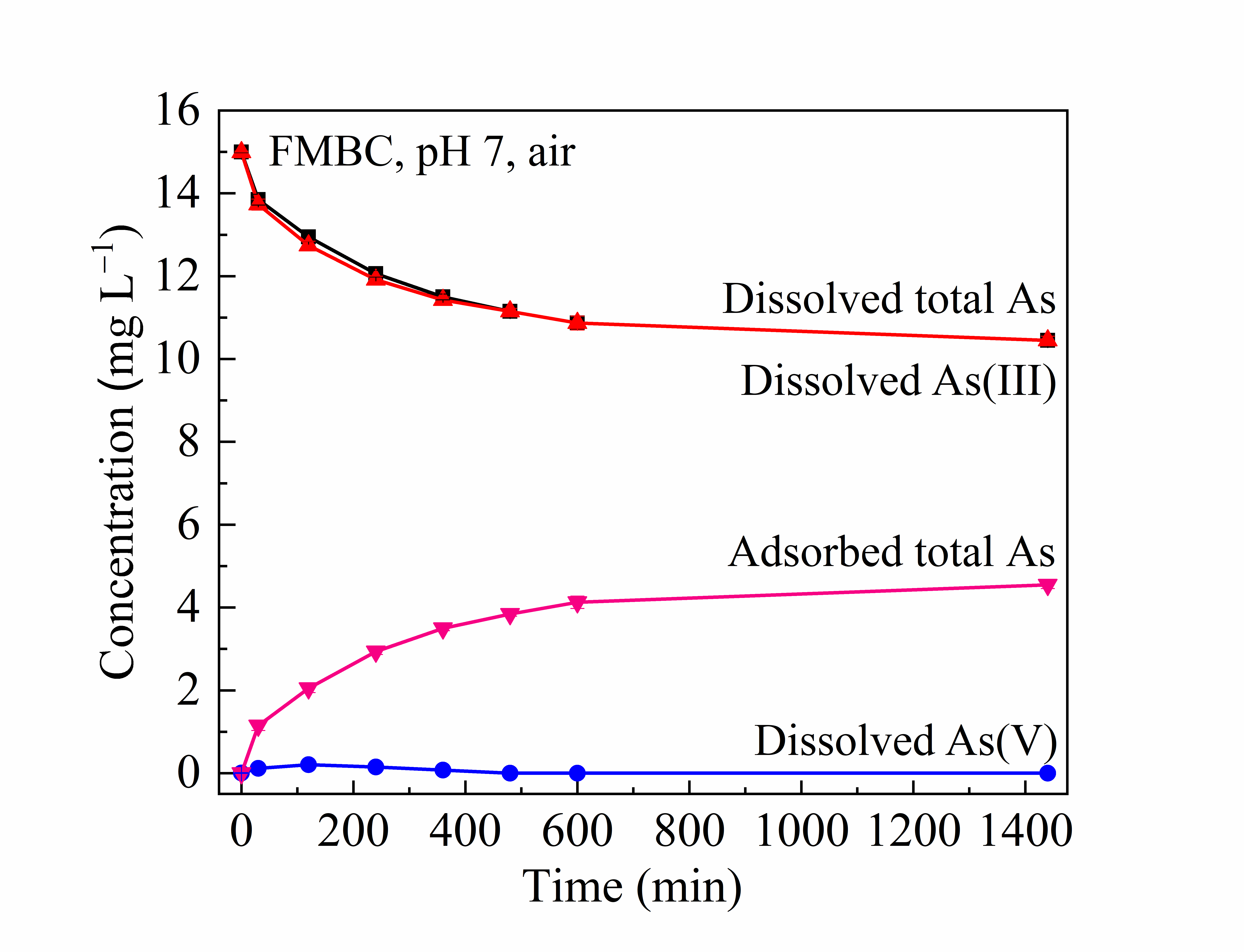


**f**


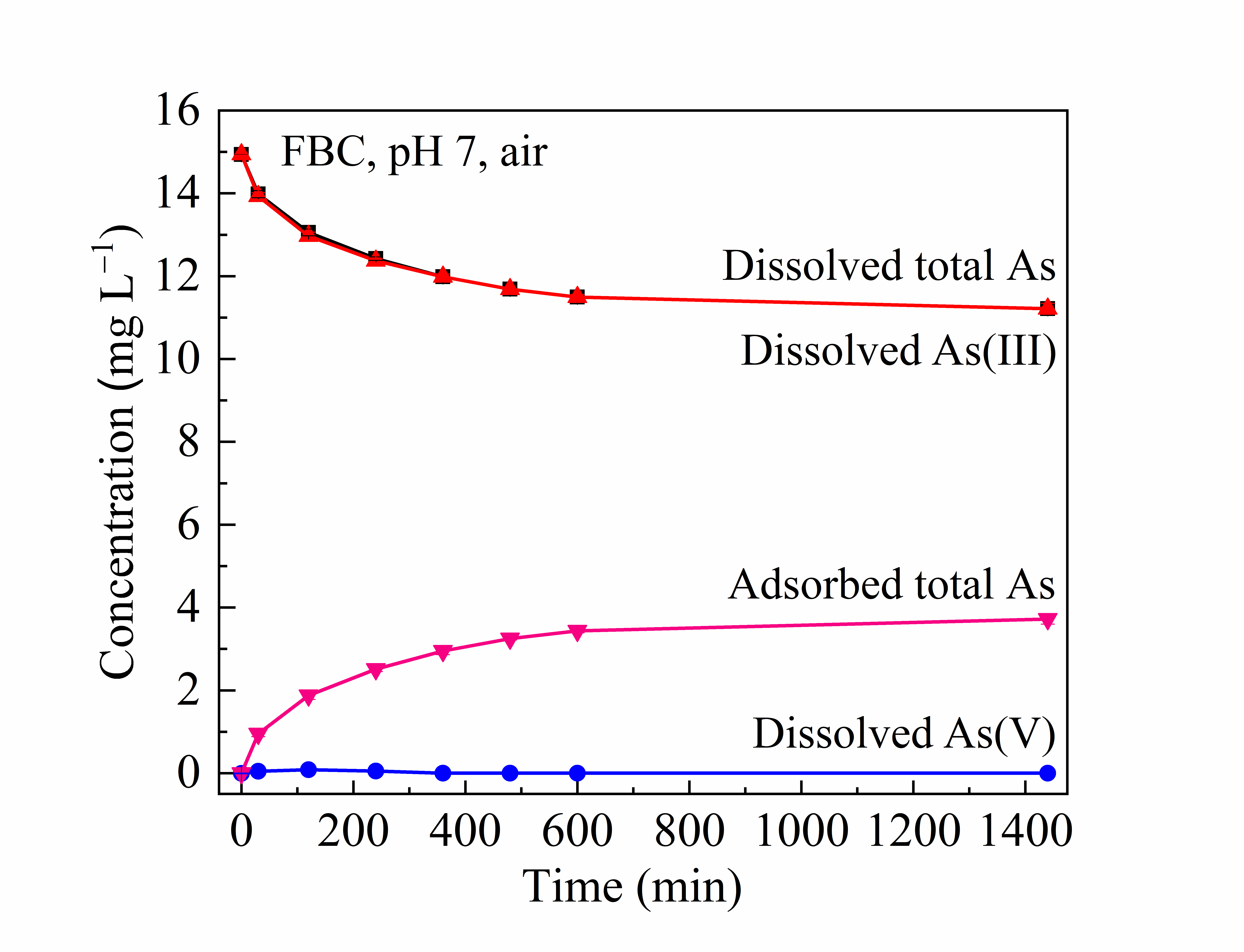


**e**


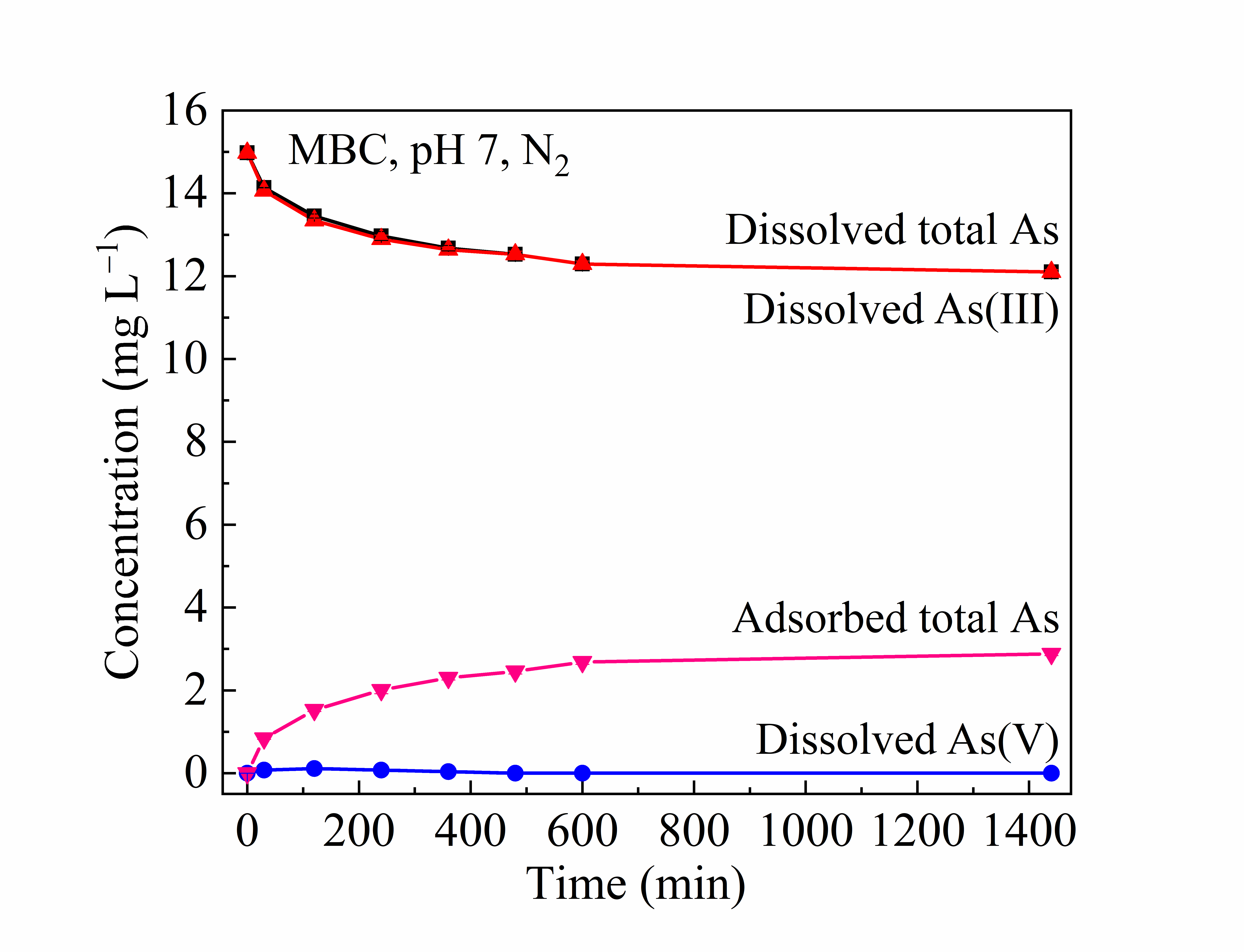


**a**


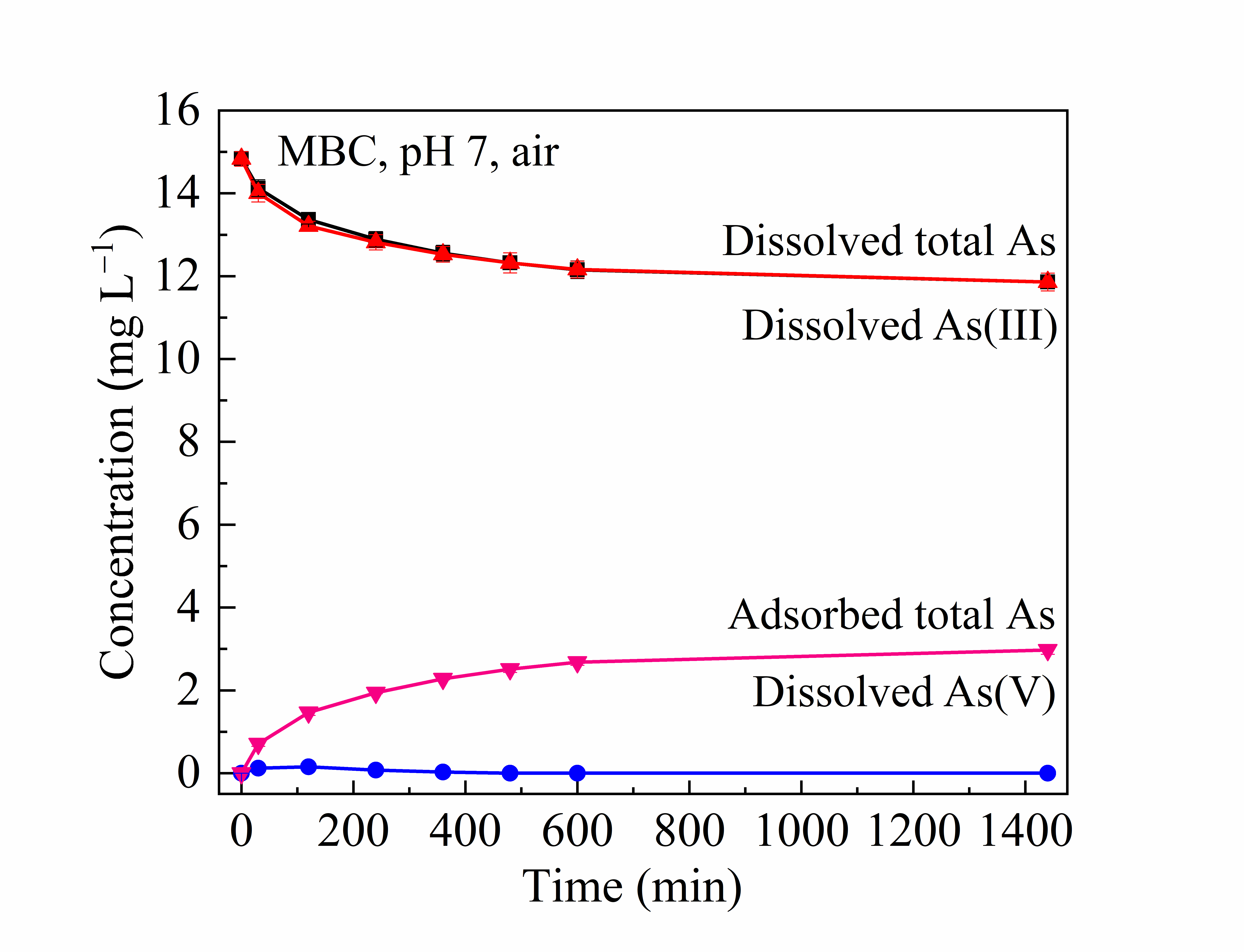


**d**

**c**


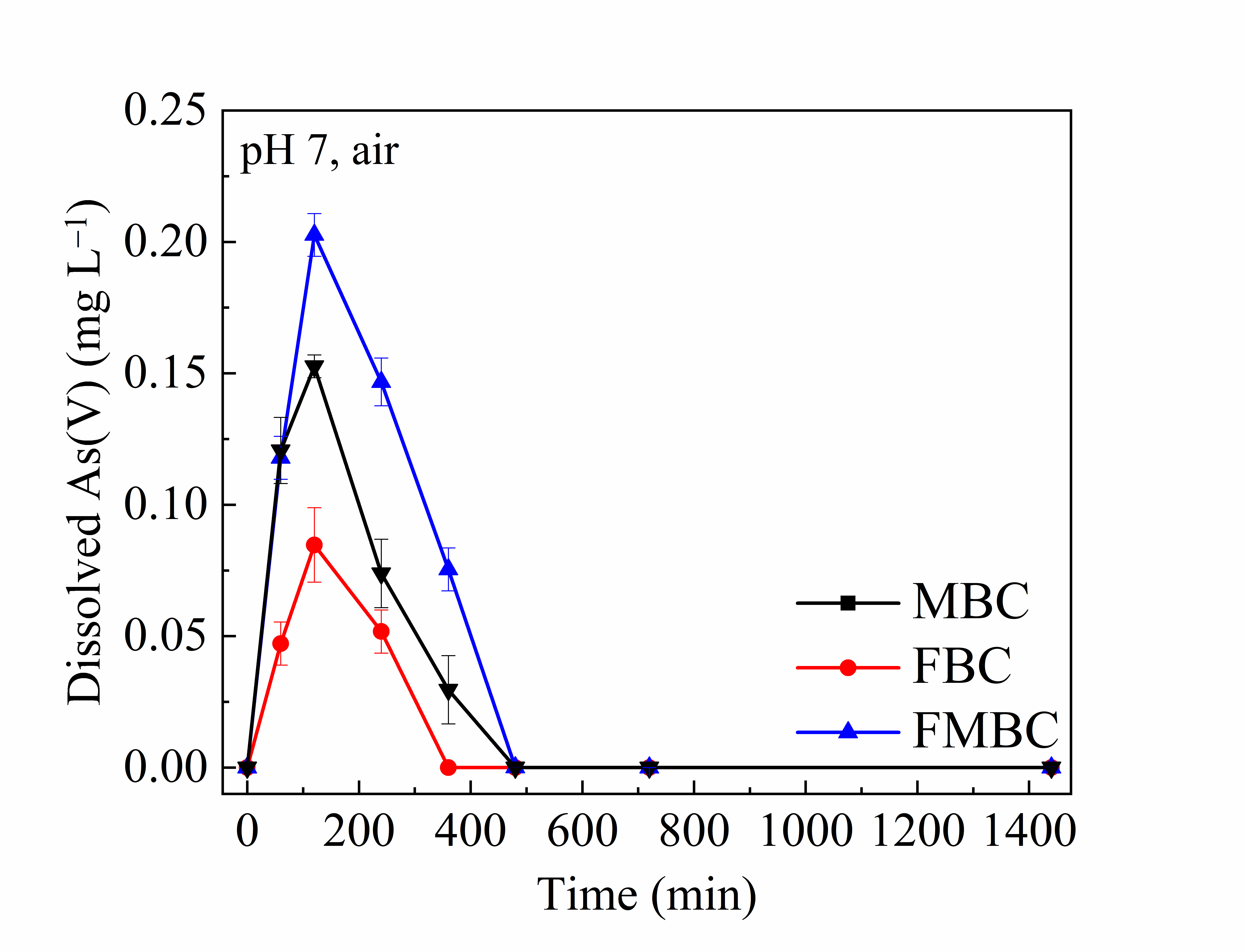


**b**


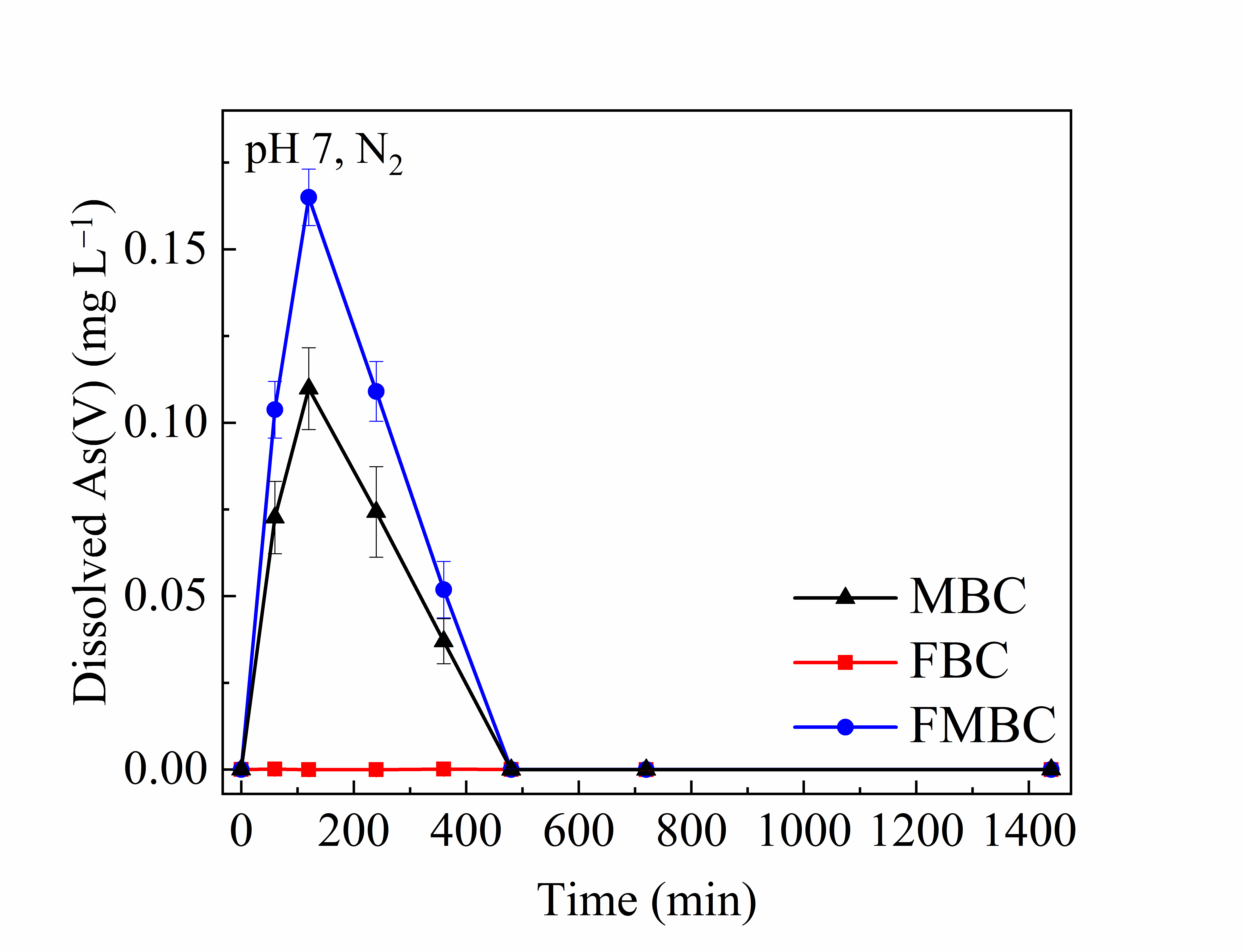


**a**


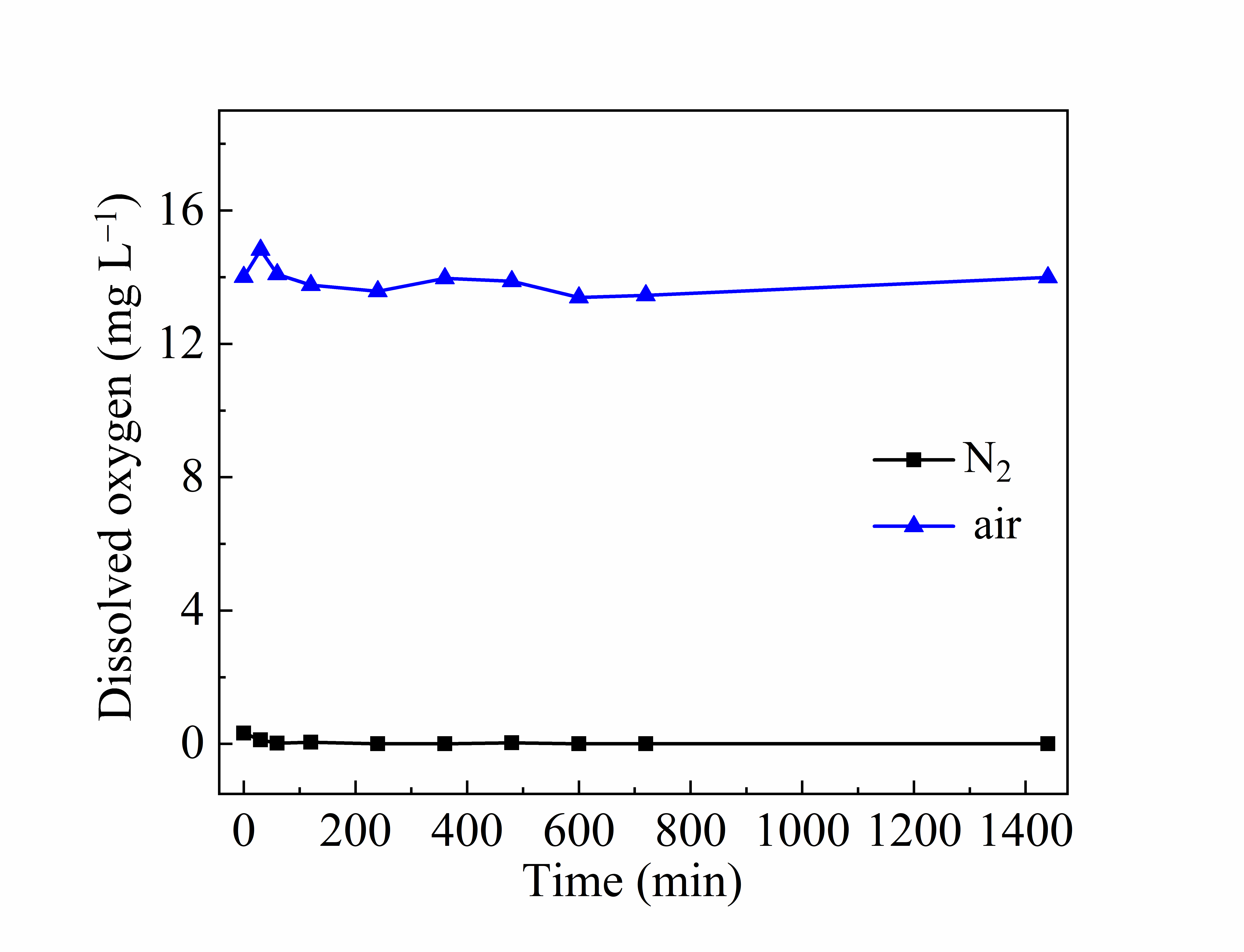


**d**


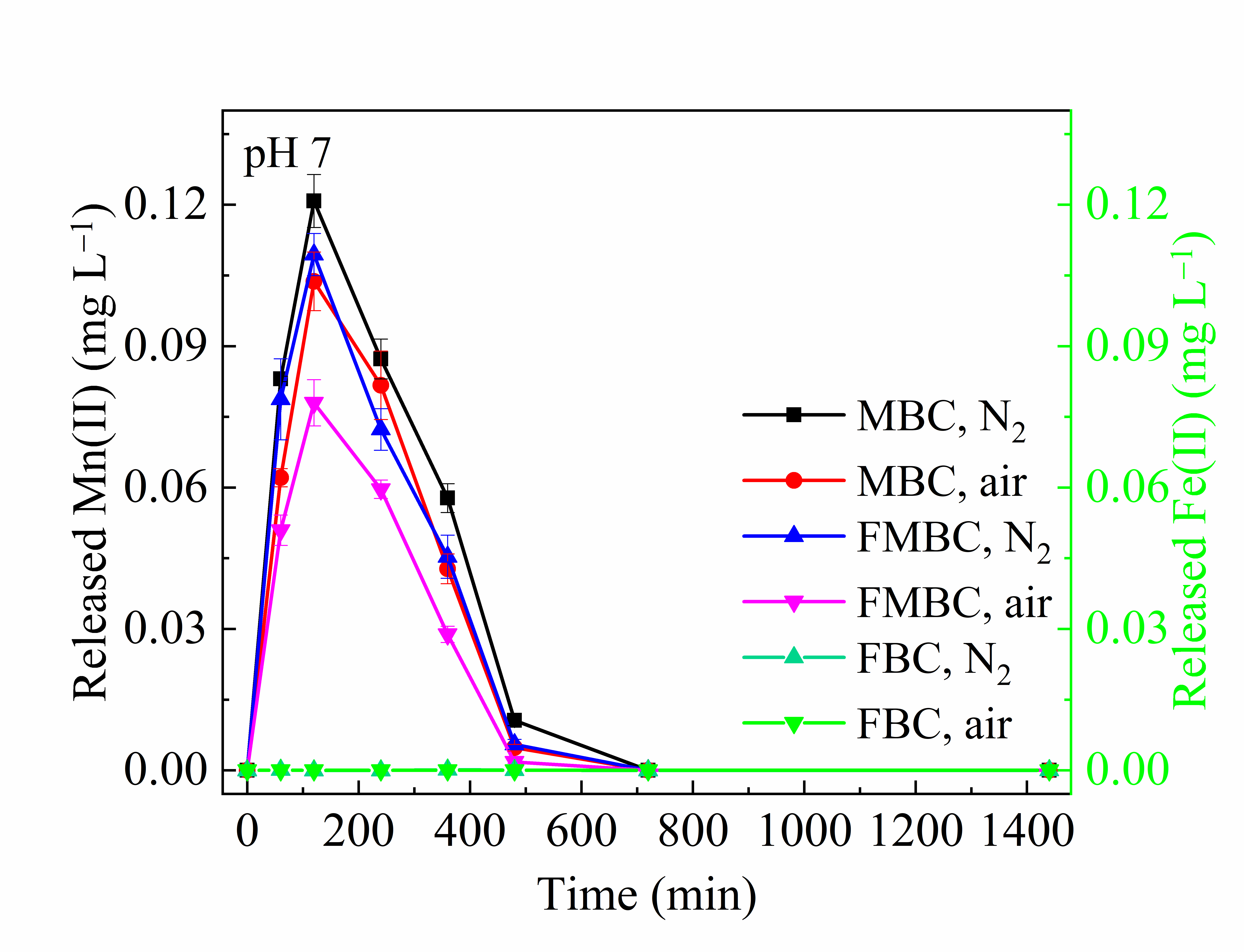


**c**

**Fig. S‎4** Concentrations of dissolved As(V) (a and b), released Mn/Fe(II) (c), and DO (d) in the reaction system containing 15 mg L^−1^ As(III) and 1 g L^−1^ of MBC, FBC and FMBC at pH 7.0 in the presence of nitrogen and air atmospheres.





**Fig. S5** Cumulative concentrations of OH^•^ under oxic conditions at pH 7.0 within 1440 min.

**

**

**Fig. S6** Effect of coexisting anions on As(III) adsorption by MBC, FBC and FMBC.

**

**

**Fig. S7** Adsorption-desorption cycles for As(III) on MBC, FBC and FMBC.


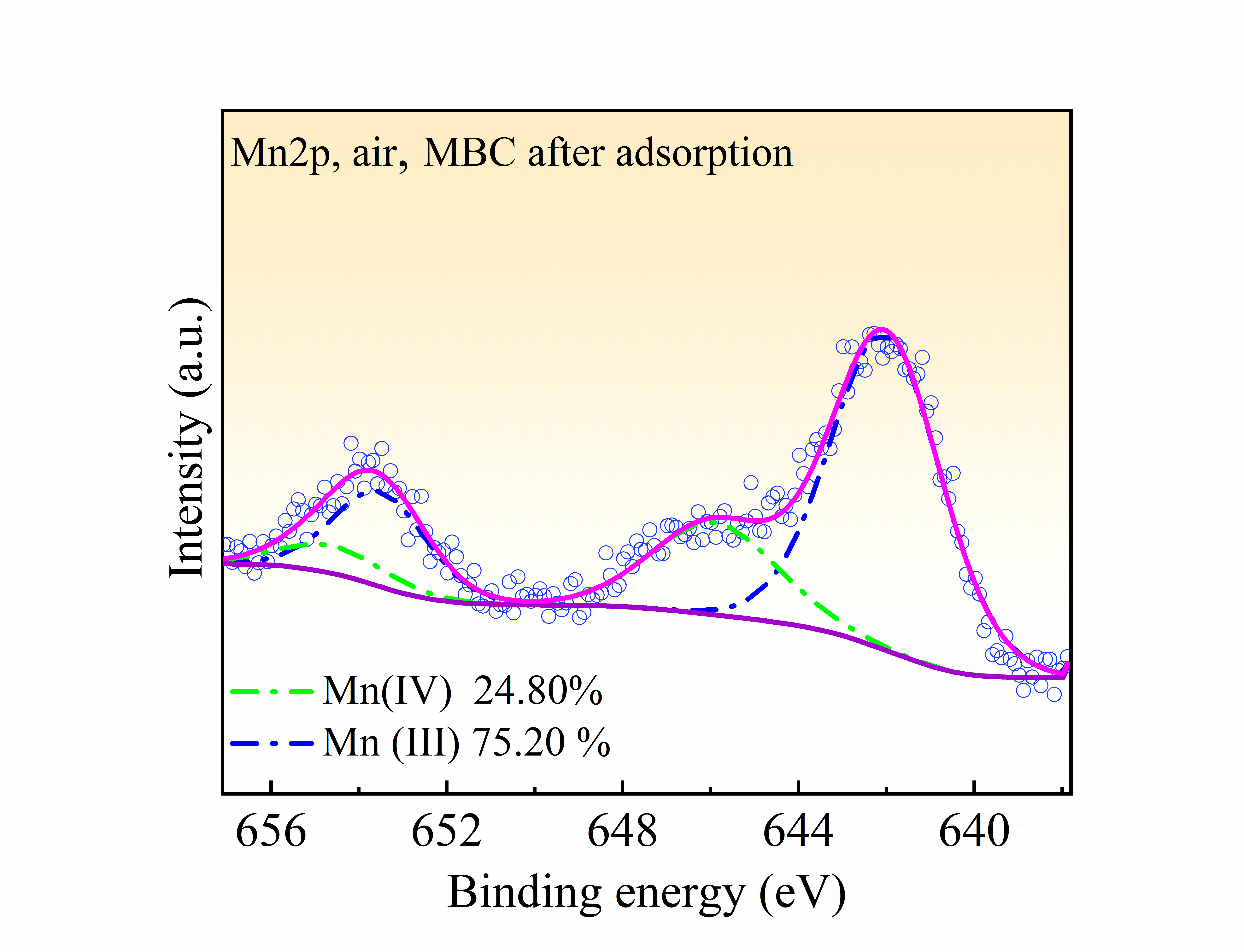


**c**


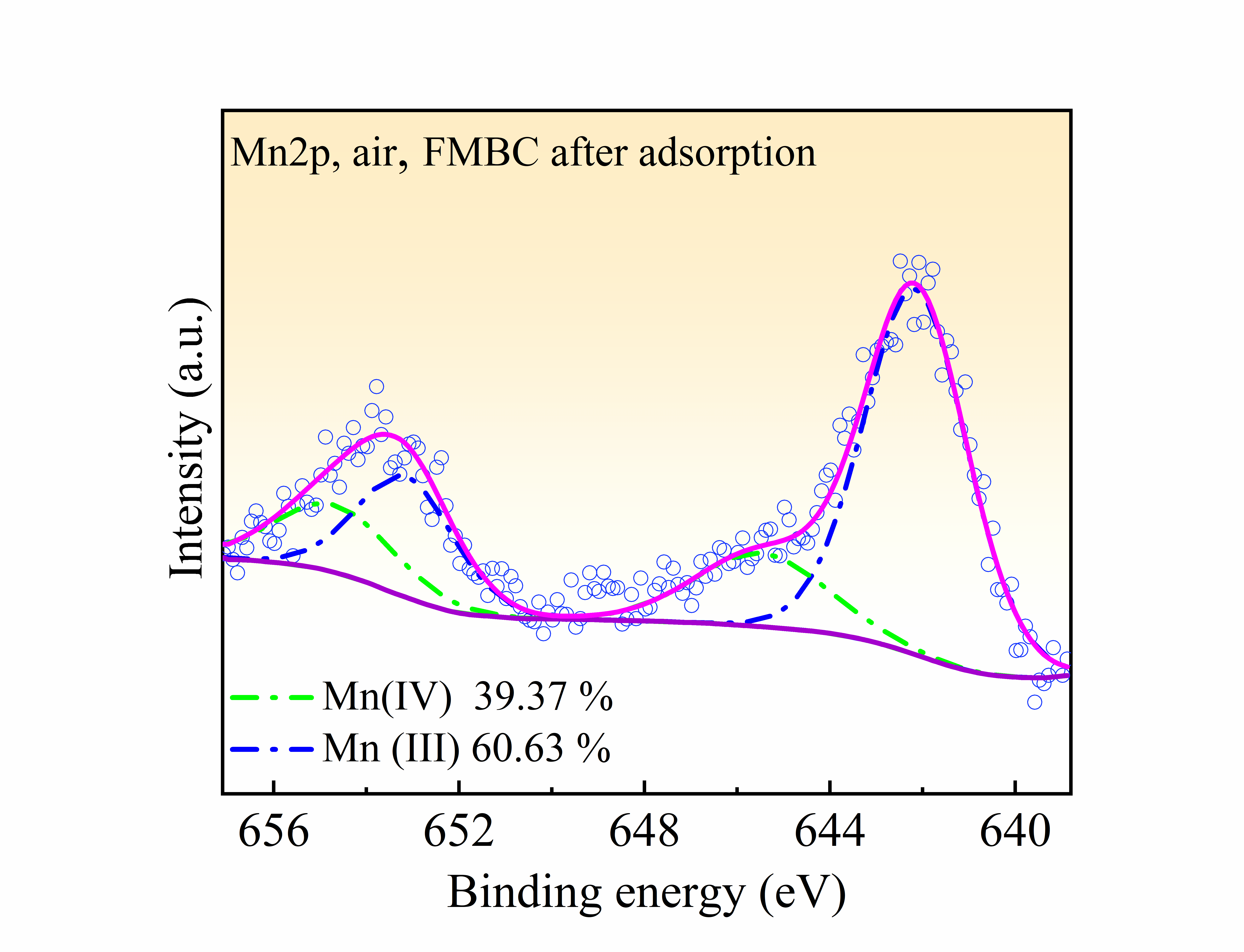


**f**


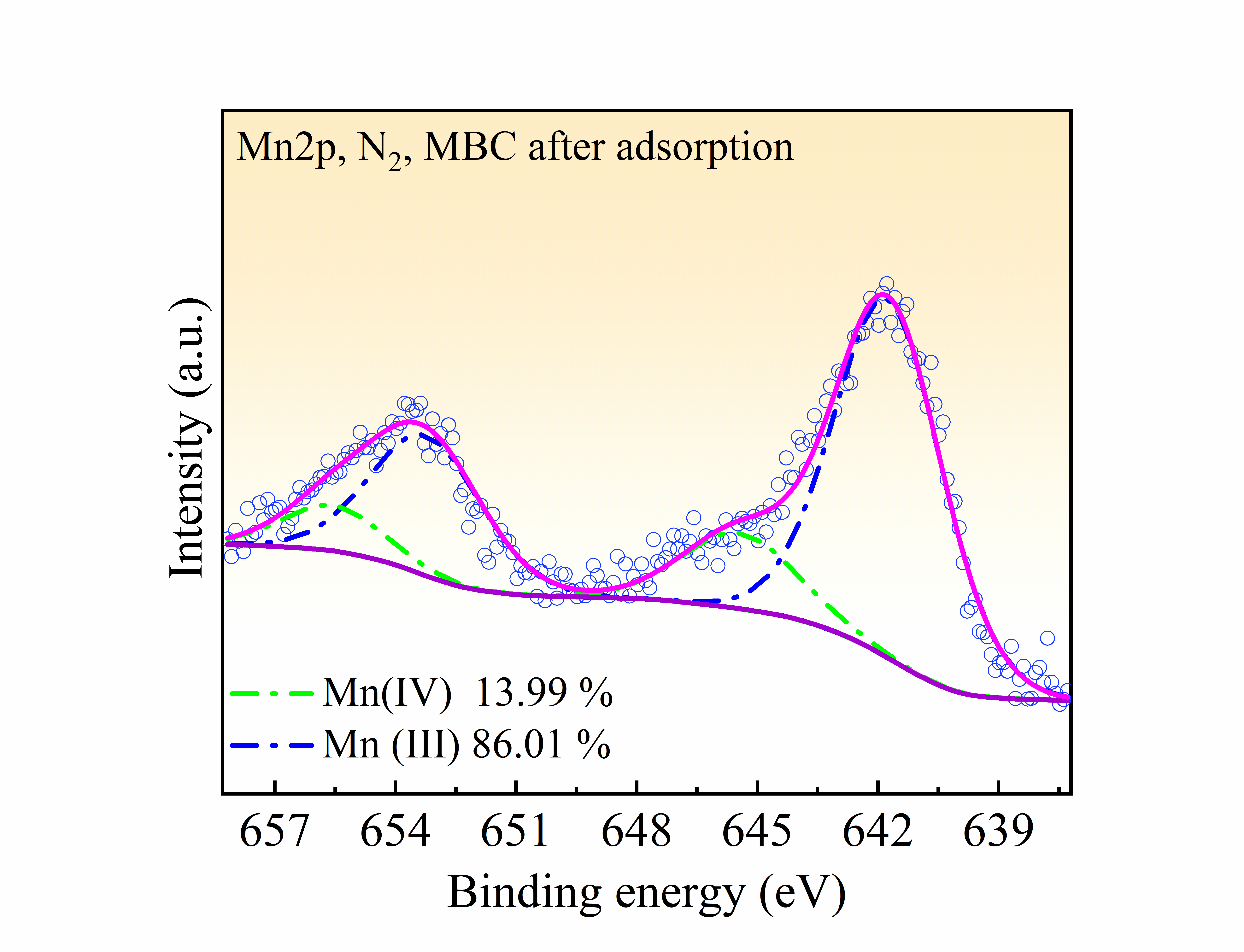


**b**


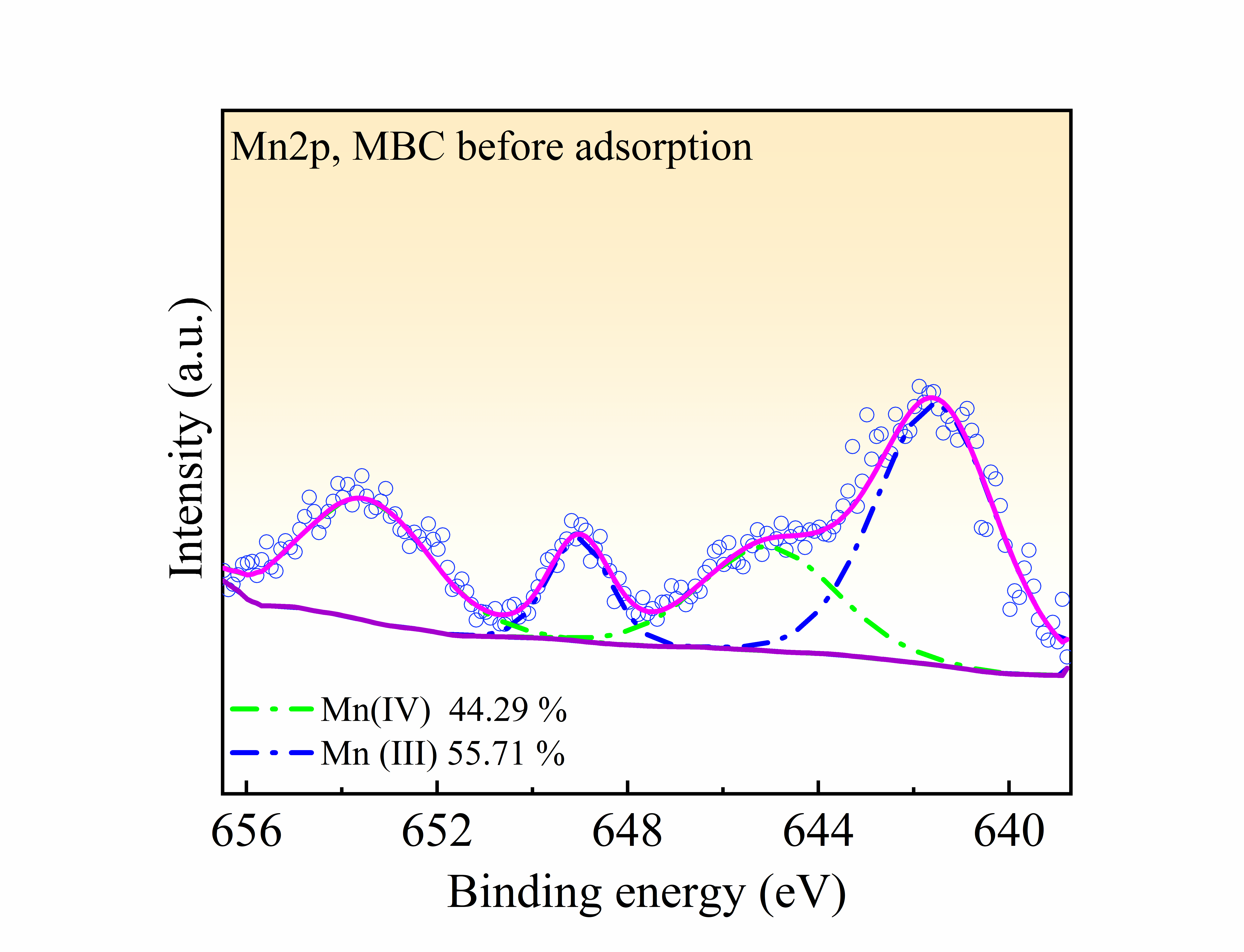


**a**


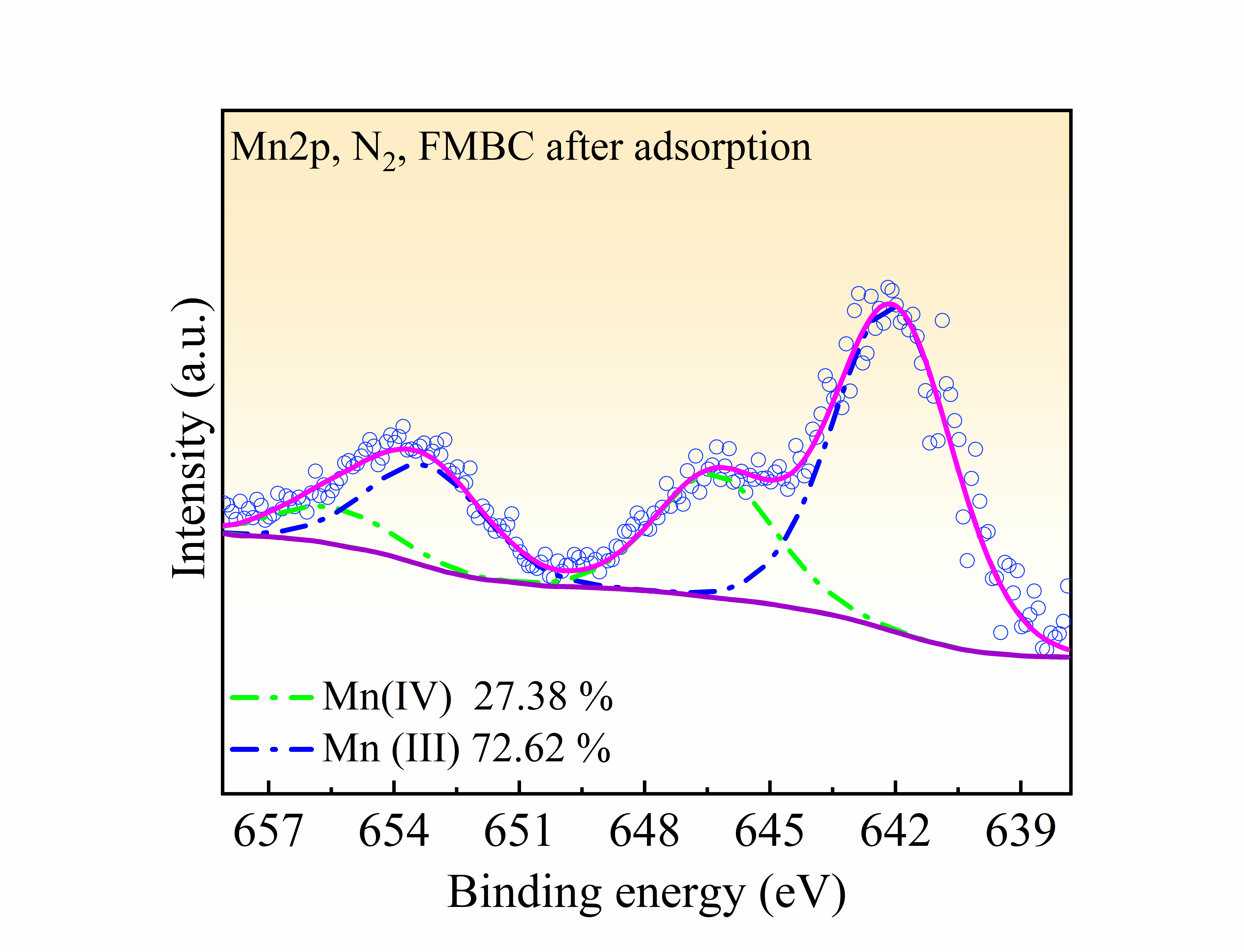


**e**


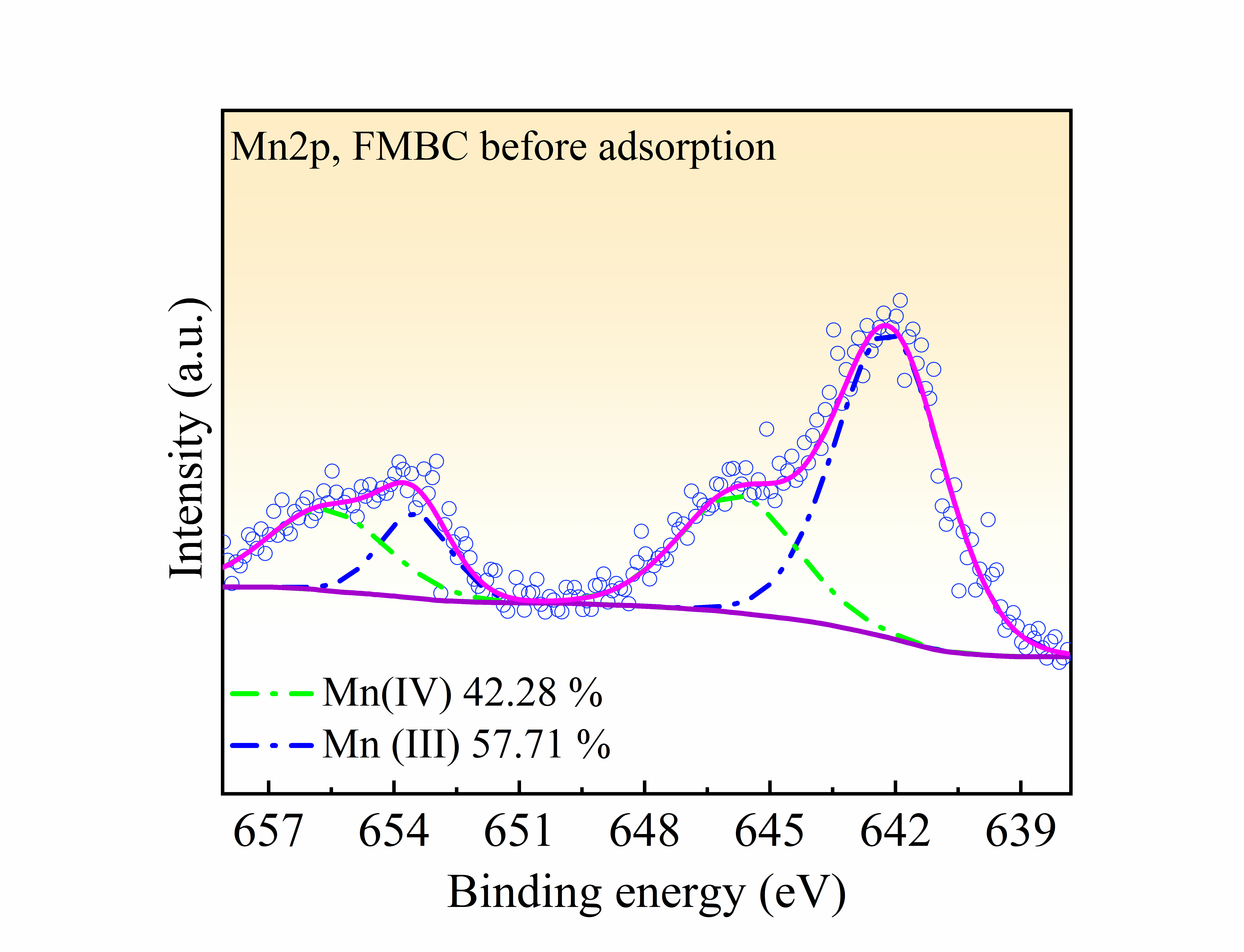


**d**

**Fig. S8** XPS Mn 2p spectra for MBC and FMBC systems before and after As(III) adsorption at pH 7.0.


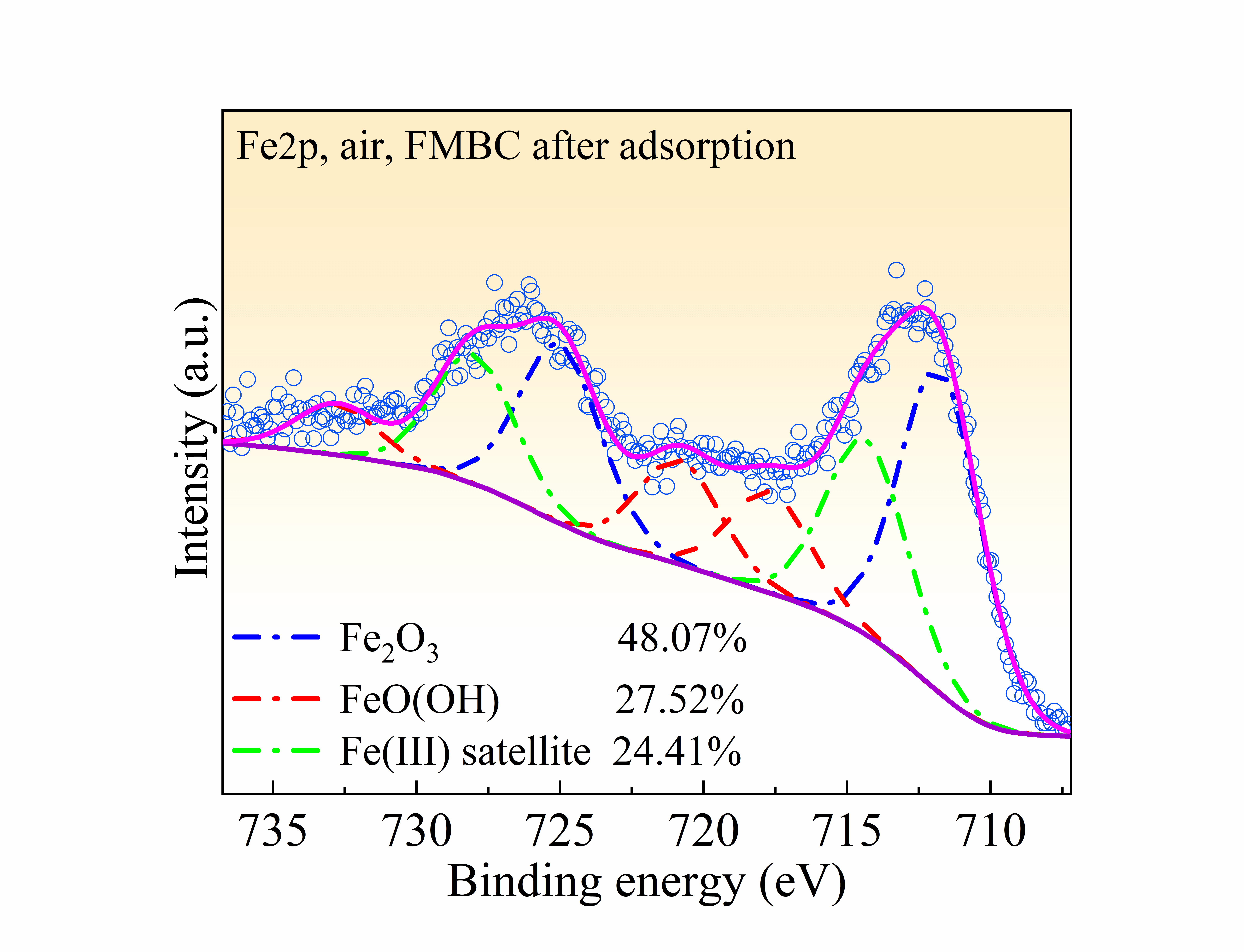


**c**


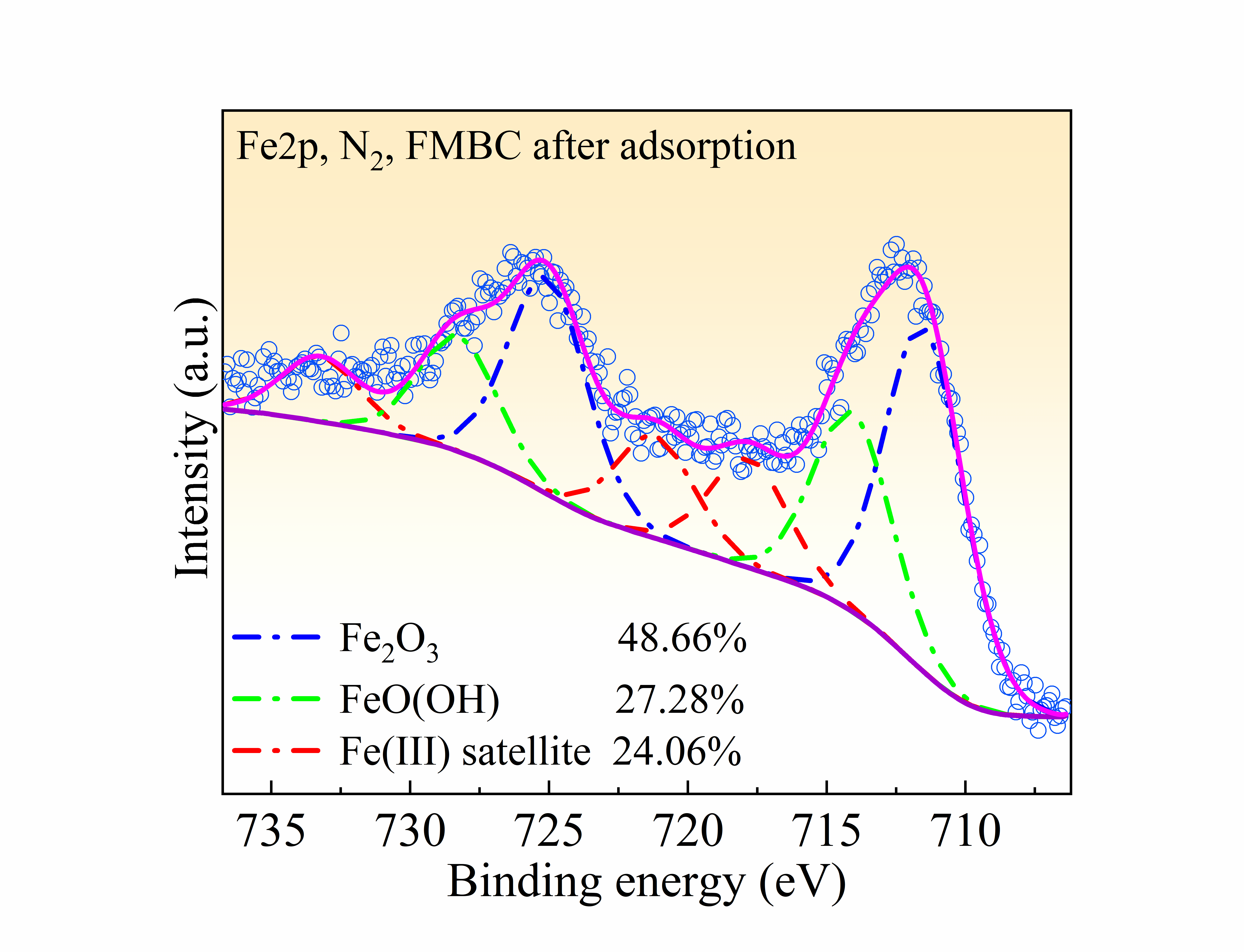


**b**


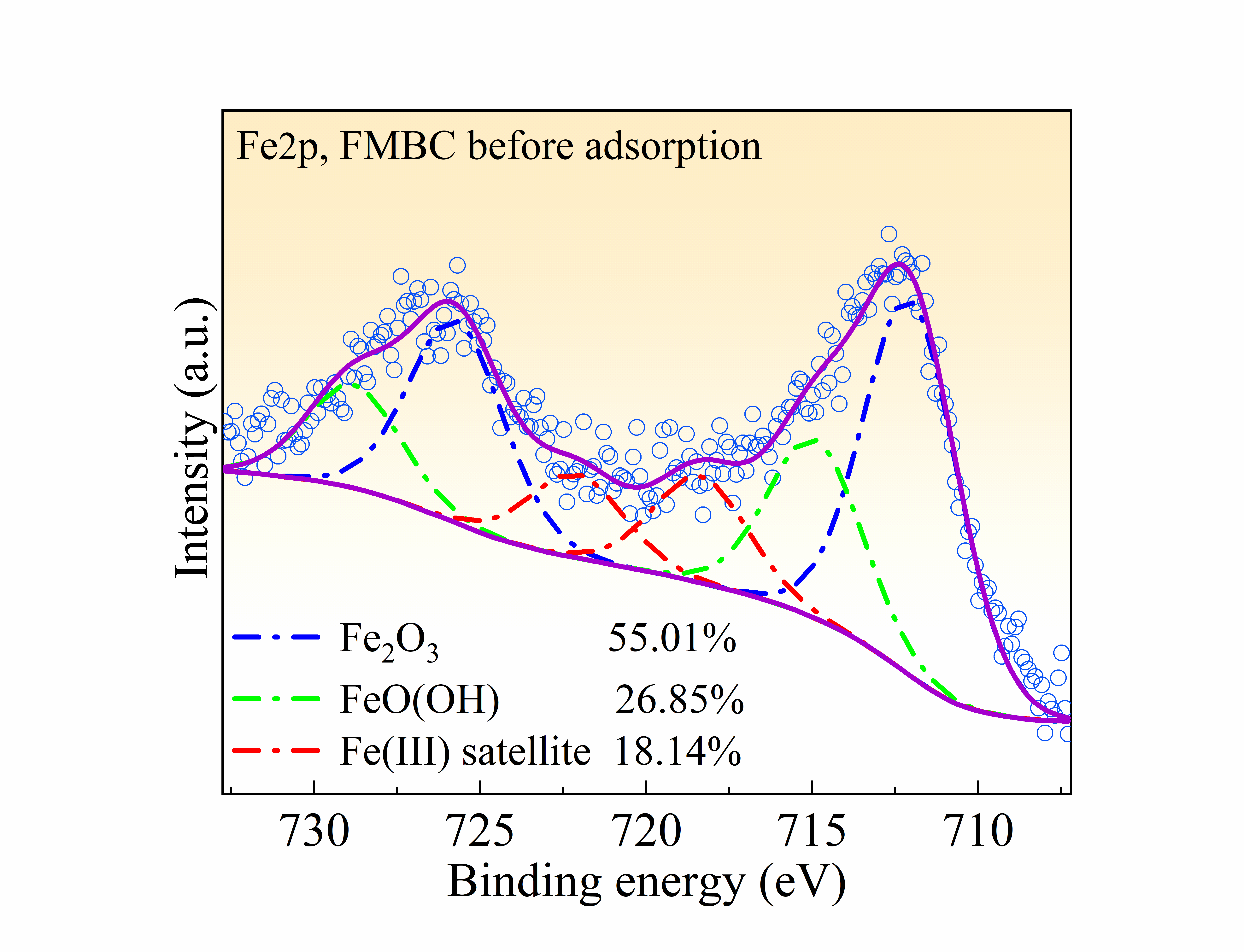


**a**


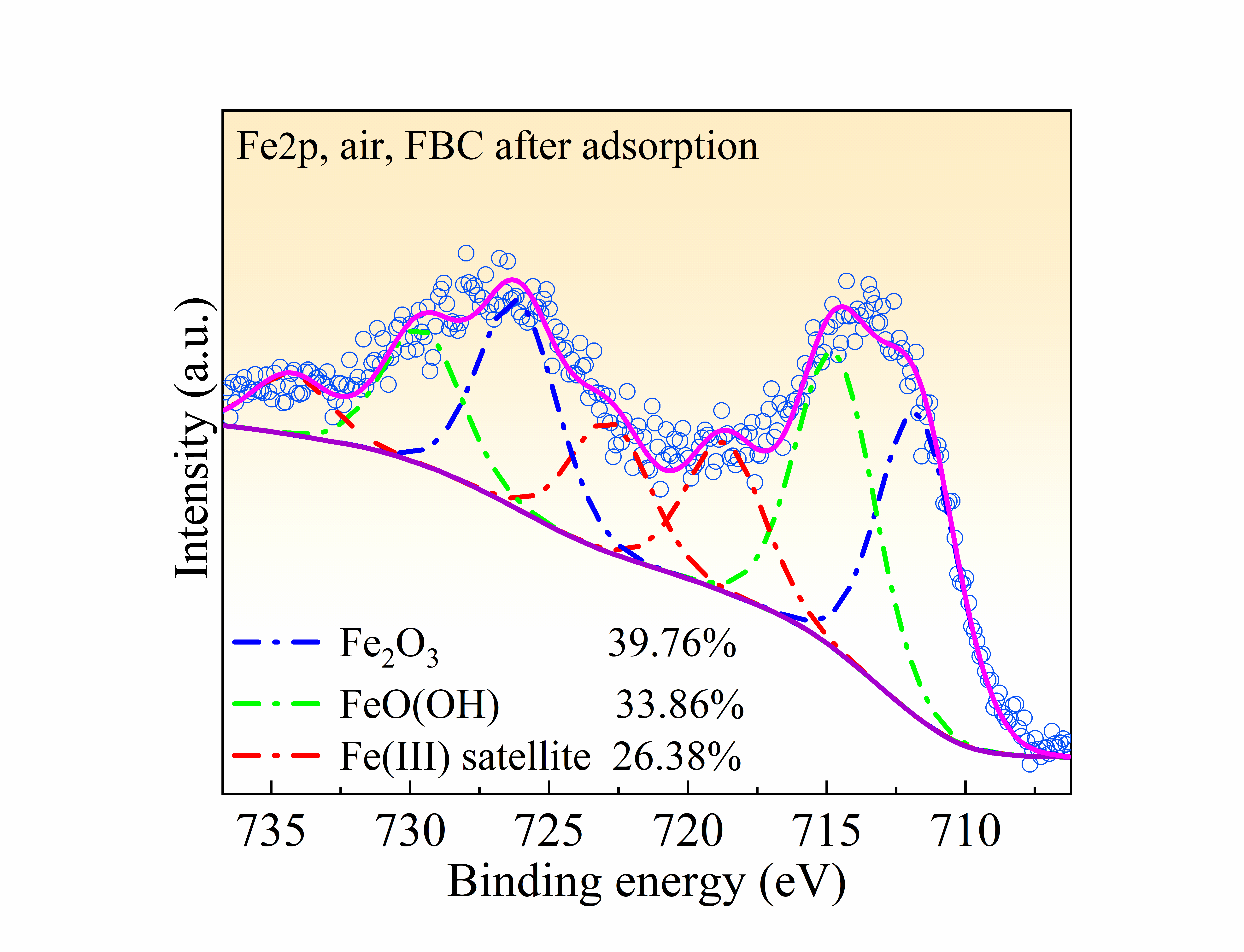


**f**


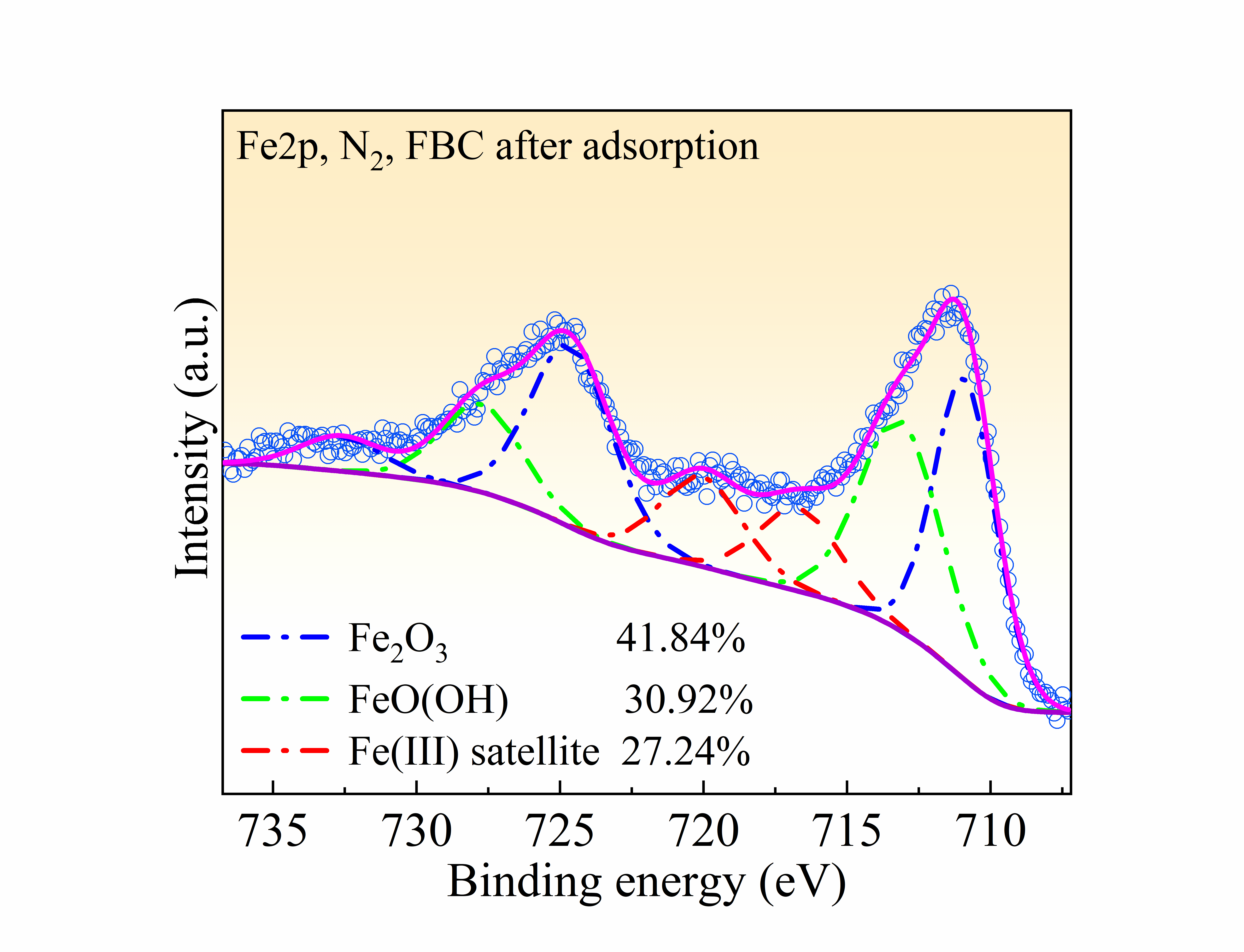


**e**


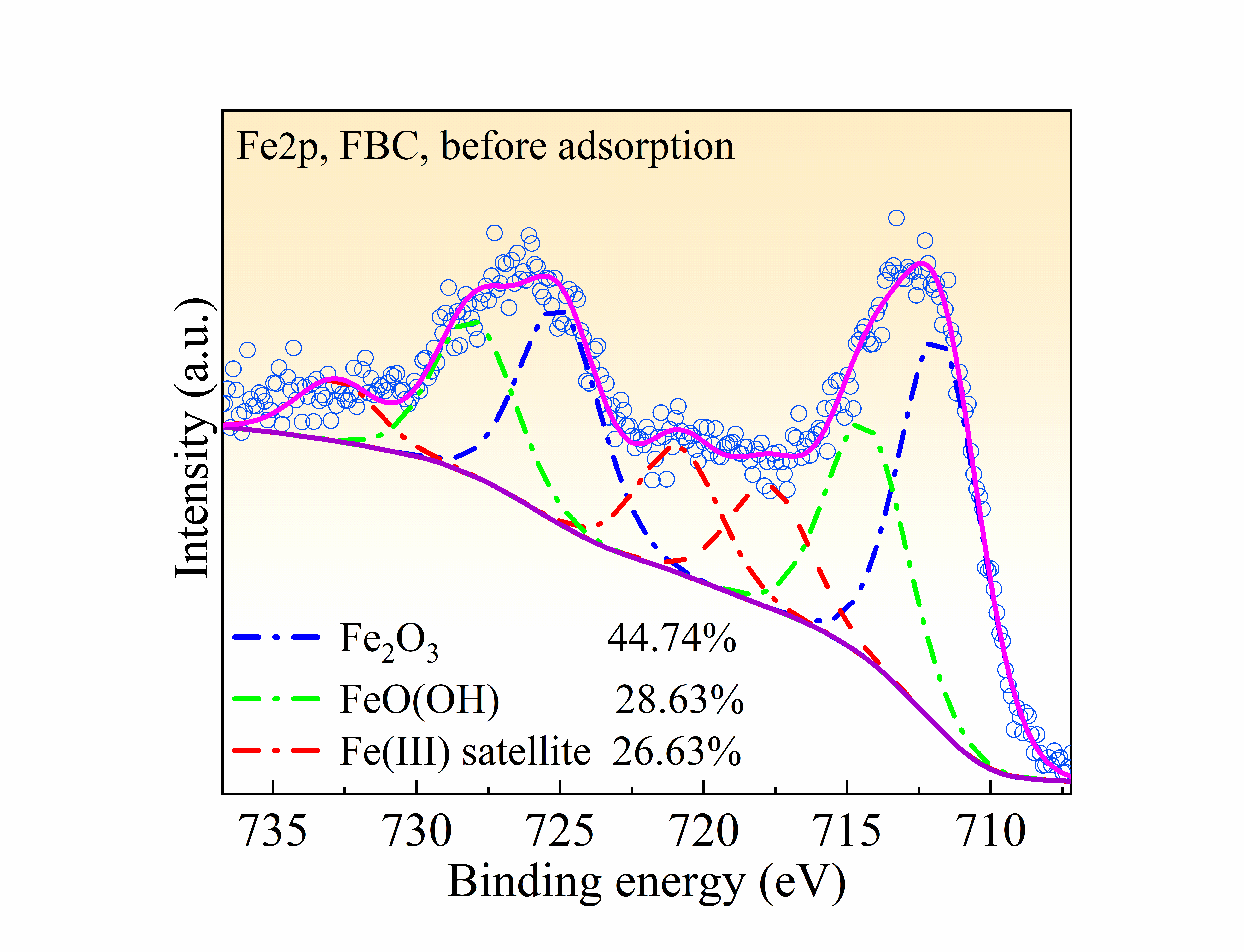


**d**

**Fig. S9** XPS Fe 2p spectra for FMBC and FBC before and after As(III) adsorption.


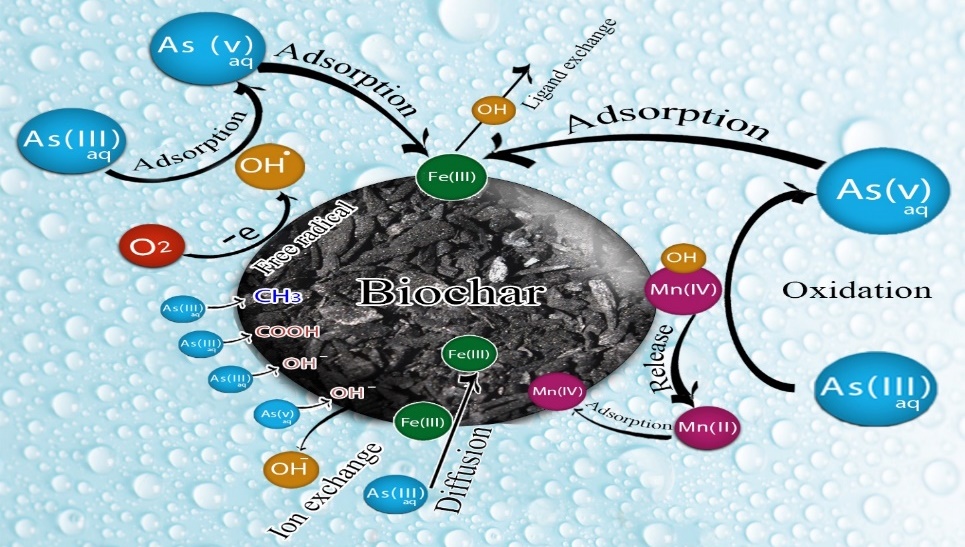


**Fig. S10** Proposed schematic diagram of the mechanisms involved in As(III/V) removal by FMBC.

**References**

1 Nesbitt, H. W. et al. XPS study of reductive dissolution of 7Å-birnessite by H_3_AsO_3_, with constraints on reaction mechanism. *Geochim. Cosmochim. Acta* **62**, 2097-2110, doi:<https://doi.org/10.1016/S0016-7037(98)00146-X> (1998).
